# The effect of vitamin supplementation on neurodevelopmental and clinical outcomes in very low birth weight and very preterm infants: A systematic review and meta-analysis

**DOI:** 10.1371/journal.pone.0327628

**Published:** 2025-07-09

**Authors:** Anja Bronnert, Peter M. Bloomfield, Lilia Delgado Páramo, Luling Lin, Frank H. Bloomfield, Barbara E. Cormack

**Affiliations:** 1 The Liggins Institute, Auckland, New Zealand; 2 Starship Child Health, Auckland, New Zealand; Saitama Medical Center: Saitama Ika Daigaku Sogo Iryo Center, JAPAN

## Abstract

**Background:**

Nutrition is vital for preterm infant development. Vitamins play key roles as cofactors and gene regulators for metabolic and immune functions and are common added components of preterm infant nutrition. However, information on how vitamins impact in-hospital and neurodevelopmental outcomes is sparse. We aimed to determine the effect of fat- and water-soluble vitamin supplementation on clinical outcomes during neonatal care and later neurodevelopment of very preterm (≤32 weeks’ gestation) and very low birth weight (≤1500 g) infants.

**Methods and findings:**

4 databases and 3 clinical trial registries were systematically searched for randomised controlled trials (RCTs). Two reviewers independently extracted data and assessed quality using the Cochrane Risk of Bias tool. Meta-analyses were conducted using a random-effect model for each vitamin subgroup. Data are presented as risk ratios [95% confidence intervals]. Of 4074 references identified, 43 studies were included in the review. Only 2 reported neurodevelopment at 2 years, and only 4 were studies of water-soluble vitamins (vitamin C, 3 studies; B_12_ and folate, 1 study). Survival free from neurodisability was not affected by supplementation of vitamin A (0.89 [0.74–1.08], n = 538, very low certainty of evidence) or vitamin D (0.76 [0.46–1.27], n = 78, very low certainty of evidence). The incidence of bronchopulmonary dysplasia was decreased by vitamins D (0.58 [0.41–0.83]) and C (0.59 [0.37–0.93]), very low certainty of evidence), retinopathy of prematurity was decreased by vitamins A (0.77 [0.61–0.98]) and E (0.10 [0.01–0.80]), very low to low certainty of evidence) and intraventricular haemorrhage was decreased by vitamin E (0.70 [0.52–0.92], moderate certainty of evidence). Culture-proven sepsis was decreased by vitamin A (0.88 [0.77–0.99], moderate certainty of evidence).

**Conclusions:**

There are few and inconclusive data on the effect of vitamin supplementation in preterm infants on later neurodevelopment. Evidence for shorter-term outcomes is mostly of low certainty. Together with substantial heterogeneity in trial design, it therefore is difficult to recommend a specific supplementation regimen.

**Registry and Registry Number:** This systematic review was prospectively registered on PROSPERO, ID CRD42023418552, available from https://www.crd.york.ac.uk/prospero/display_record.php?ID=CRD42023418552

## Background

Healthy human pregnancies reach term at 37 to under 42 weeks. Infants born before term pregnancy are referred to as preterm. On average, preterm birth affects around 10% of global live births [1]. Of these, around 10% are born very preterm (VP) at less than 32 weeks of gestation, usually coinciding with a lower birth weight [2]. Neonatal care for very low birth weight (< 1500 g, VLBW) and VP infants has seen considerable achievements in recent decades. Survival rates have increased, as has survival free from major morbidity [2–4]. However, care for this most vulnerable group of infants remains challenging. Preterm birth means missing out on weeks or months of crucial growth processes in *utero* and entering the world with immature metabolic, immune, and organ development [5]. Incidence of neurodisability and sub-optimal health outcomes later in life are considerably higher than in full-term infants [4,6–10]. Neonatal complications, including increased inflammation and infection, contribute to compromised brain maturation alongside impaired nutrition [6]. VP infants are born in a state of malnutrition with little or no subcutaneous fat, glycogen, mineral, and vitamin stores. Adequate nutrition as soon as possible after birth is vital to maintain growth, support immune and metabolic function, and ensure optimal neurodevelopment [11,12]. However, the actual nutritional requirements of VP and VLBW infants are not known, especially their vitamin requirements.

Vitamins are essential nutrients that are required in small quantities but cannot be synthesised by the human body, either at all or in sufficient quantities. Provitamins are precursors that are converted to active compounds by metabolic processes in the body, such as the cleavage of beta-carotene to vitamin A. Vitamins may occur in various vitamers, similar forms with related functions, such as the various forms of tocopherols and tocotrienols of vitamin E. Active vitamins and their vitamers have diverse biochemical functions as co-factors for enzymatic reactions or regulators of gene expression in the energy metabolism and immune system. Therefore, deficiency can have serious to fatal consequences. The quantity of vitamins provided during neonatal intensive care, first through parenteral and later enteral nutrition, is based on estimations of requirements, and little is known about whether supplementation can improve development and clinical outcomes [11,13]. A recent systematic review of 27 guidelines on parenteral and enteral nutrition of preterm infants found the recommendations for fat- and water-soluble vitamin intake inconsistent and the evidence presented in the guidelines very weak. Intake recommendations vary between guidelines, and the recommended intake ranges for some vitamins are very wide [14]. The variation found in this systematic review of guidelines reflects the lack of conclusive research regarding dosages of vitamin supplementation and infants’ vitamin status that support improved health outcomes, leading to disparities in practice that may result in vitamin intake for preterm babies exceeding or falling short of their requirements [15].

## Objective

We therefore undertook a systematic review to assess the effect of supplementation with any vitamin in VLBW and VP infants on survival free from neurodisability at 2 years or beyond, as well as its impact on growth, neonatal morbidities, and death before discharge from initial hospitalisation after birth.

## Methods

This systematic review was reported according to Preferred Reporting Items for Systematic Reviews and Meta-Analyses (PRISMA) guidelines and registered prospectively in PROSPERO (registration number CRD42023418552).

### Types of studies

Randomised controlled trials (RCTs), quasi-RCTs, and cluster-RCTs were included. Cross-over RCTs were not included as their design is not suitable for measuring long-term outcomes. Abstracts that provided all necessary information were eligible for inclusion. Relevant ongoing and unpublished recent clinical trials were identified, and lead investigators were approached for data. Non-randomised trials were excluded.

### Types of participants

Infants born at a VLBW (≤ 1500 g) or VP (≤ 32 weeks’ gestational age) of all sexes and ethnicities were included. While the World Health Organization’s definition of VLBW is < 1500 g [16], a slightly broader weight limit was chosen for this systematic review to account for different definitions chosen by study authors.

### Intervention

Any interventional study that aimed to increase the intake of any fat-soluble or water-soluble vitamin or their respective provitamins, reporting on one or more of our primary or secondary outcome measures, was included. The route of administration could be enteral, intravenous, intramuscular, or a combination. The intervention could commence at any time during the initial hospitalisation after birth and be concluded at the time of discharge. We included studies that compared an intervention with a placebo, studies with two arms of interventions of the same type but with different doses, with or without a placebo arm, and studies that gave more than one of the relevant vitamins in combination as a multivitamin supplementation. Differences in the intervention were categorised as defined in the methods of the studies.

Standard feeding solutions for preterm infants usually include vitamins. This was not regarded as vitamin supplementation. Any extra vitamin administration on top of standard nutrition was regarded as supplementation. Studies in a wider population (e.g., all preterm births) that conducted subgroup analysis on the population of interest for this systematic review were eligible, provided the necessary subgroup data were available or could be provided by the authors upon request. We excluded studies that investigated the effect of supplementation after hospital discharge, studies that did not report on any clinical outcomes, and studies comparing different modes, timing, or designs of vitamin administration.

### Outcome measures

#### Primary outcome.

The primary outcome was survival free from neurodisability at 2 years or beyond. Neurodisability was defined using, but not limited to, cognitive, language, and motor impairment, blindness, deafness, cerebral palsy, and measures of brain growth.

#### Secondary outcomes.

Secondary outcomes were components of primary outcomes, as well as neonatal morbidities, including retinopathy of prematurity (ROP), intraventricular haemorrhage (IVH), bronchopulmonary dysplasia (BPD) or chronic lung disease, necrotising enterocolitis (NEC), sepsis, anaemia of prematurity, growth measures, and death.

Subcategories were added for some outcomes to increase the precision of the analysis. ROP was split into all ROP and severe ROP, defined as ROP stage ≥3 or requiring treatment. IVH was split into all IVH and severe IVH, defined as IVH grades 3 and 4. Only studies that commenced supplementation within the first 24 hours after birth were included for this outcome, as onset of IVH after the first few days after birth is rare. For BPD, only data from studies that reported BPD as a need for oxygen at 28 days or 36 weeks postmenstrual age (PMA) or based on radiological requirements were included. If data for both time points were available, the 36-week timepoint was used for the meta-analysis. Other definitions, such as total days on ventilation, were not included. Sepsis was split into culture-proven sepsis only and proven and suspected sepsis combined.

### Search methods and identification of studies

A search strategy was developed with a librarian to search the databases CENTRAL, Embase, Ovid MEDLINE, and CINAHL on 01.05.2023 and 02.05.2023. In addition, a search for registered trials was conducted on ClinicalTrials.gov, The WHO International Clinical Trials Registry Platform, and The Australian and New Zealand Clinical Trials Registry on the same dates. Searches were rerun on 14.12.2023 and 28.05.2024. The search strategies can be found in S1 File. The same strategy was used for all databases and registries; search terms and operators were adjusted where necessary.

Ongoing studies were identified and documented to evaluate for future inclusion in review updates. The search was not limited to any language or date of publication; however, only English search terms were used. Search terms accounted for both English and American spelling. In addition, all citations in the included studies were screened for publications that might have been missed in the search.

### Data collection and analysis

#### Selection of studies.

All articles identified by the search strategy were imported into Covidence [17], a systematic review screening tool, to collect search results and remove duplicates. The titles and abstracts of all references were screened by two authors (AB and LDP) independently. The full text for all relevant studies was further evaluated according to the inclusion and exclusion criteria. Discrepancies were resolved by bilateral discussion first and, if necessary, with a third author (LL).

#### Data extraction and management.

All relevant data were extracted into a prespecified extraction spreadsheet, including administrative information, study methodology, outcome data, and information to assess the risk of bias. The extraction was completed by two authors independently (AB and PMB) and any discrepancies were discussed and resolved after completion. Quantitative data was entered into RevMan 5.4 [18] for meta-analysis.

#### Assessment of risk of bias.

All included studies were assessed for risk of bias (ROB) by two review authors (AB and PMB) independently using the Cochrane ROB 2 tool [19]. ROB 2 assesses the risk of bias on an outcome level. The risk of bias is assessed in five domains: randomisation and allocation concealment, deviations from the intended intervention, missing outcome data, outcome measurement, and reporting bias. All outcomes of the included studies were categorised into low risk of bias, high risk of bias, or some concerns.

#### Process for missing data.

In the case of important missing data, authors were contacted to obtain the necessary information. Authors of studies published before the year 2000 were not contacted. If no response was received, missing methodological data was reflected in the risk of bias assessment. If there was no relevant outcome data reported, the study was excluded.

#### Certainty of evidence.

The “Grading of Recommendations, Assessment, Development and Evaluation” (GRADE) [20] approach was used to assess the certainty of the evidence of the following outcomes: neurodevelopment and survival free of neurodisability at 2 years’ corrected age or beyond; diagnosis of ROP during initial hospitalisation after birth; diagnosis of IVH or other serious bleeding during initial hospitalisation after birth; diagnosis of BPD or chronic lung disease during initial hospitalisation after birth; diagnosis of NEC during initial hospitalisation after birth; diagnosis of sepsis during initial hospitalisation after birth; death during initial hospitalisation after birth.

This was assessed by two review authors (AB and PMB) independently. The certainty of evidence was summarised in a Summary of Findings Table based on the GRADEpro Guideline Development Tool.

#### Data analysis.

Data analysis was done using RevMan 5.4. Separate analyses were conducted to determine the effect of each single vitamin or vitamin combination. Due to considerable heterogeneity between the included studies regarding intervention, participants, and methodology, among other factors, if more than one study was included, a random-effects meta-analysis was used. Pre-specified subgroup analyses were conducted where data were available to explain heterogeneity in outcomes, as well as sensitivity analysis by excluding studies at high risk of bias.

For studies with more than two arms, the control or lowest dose group was split in two and double-entered into the meta-analysis to be compared with the remaining two groups. This was necessary to maintain the correct number of participants in the meta-analysis, as opposed to entering the full control group into the meta-analysis twice to be compared with both intervention arms.

### Effect measures of outcomes

For dichotomous data, the number of outcome events in the control vs the intervention group was used to calculate risk ratios (RRs) and risk differences (RDs) with a 95% confidence interval (CI). If there was a statistically significant reduction or increase in the RD after the intervention, additional calculations were done for the number needed to treat (NNT) for an additional beneficial or harmful outcome. The mean difference (MD) with a 95% CI was calculated for continuous outcomes.

#### Assessment of heterogeneity.

The I^2^ statistic was used to quantify inconsistency among the included studies, and the Chi^2^ test was used to indicate whether any heterogeneity was significant (p < 0.1). Where substantial heterogeneity was detected, possible causes were explored by looking at methods and, if possible, conducting subgroup analyses.

### Subgroup analysis

Subgroup analyses were planned for the following subgroups: extremely low birth weight (ELBW) ≤ 1000 g; extremely preterm (EP) ≤ 28 weeks; mode of administration of the supplementation: intravenous, intramuscular, enteral; supplementation regimen: e.g., daily, 3 times per week, single dose; ethnicity; country of birth: High and upper-middle-income countries vs. low and lower-middle-income countries according to the 2024 World Bank classification [21].

## Results

### Search results

**Fig 1:**
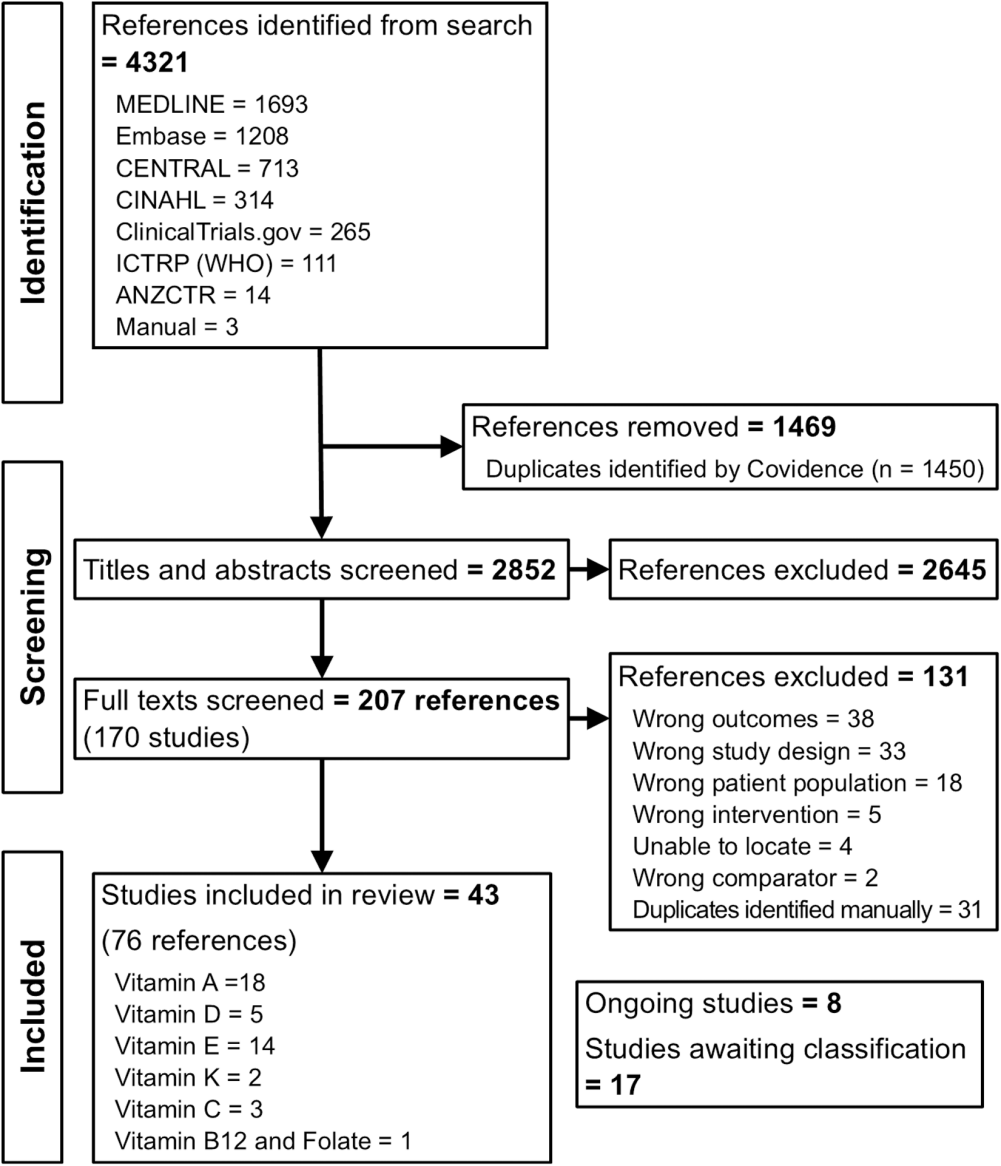
PRISMA diagram. ICTRP, International Clinical Trials Registry Platform; WHO, World Health Organization; ANZCTR, Australian New Zealand Clinical Trials Registry.

Fig 1 shows the study screening and selection process. The search resulted in 4321 references, of which 1469 were automatically removed as duplicates by Covidence. After reviewing 2852 abstracts, 207 full-text references reporting on 170 studies were screened. An overview of reports resulting from the search and reasons for exclusion are presented in S1 Table. Articles not written in English were translated by native speakers. After screening the reference lists of included studies to identify missing records, 3 records were added manually (Hittner 1984 [22], Bental 1994 [23], and Schaffer 1988 [24]). This resulted in 76 references reporting on 43 studies included in the qualitative synthesis. Eighteen studies with 3350 infants investigated vitamin A supplementation; 5 studies with 387 infants, vitamin D; 14 studies with 1874 infants, vitamin E; 2 studies with 124 infants, vitamin K; 3 studies with 251 infants, vitamin C; and 1 study with 64 infants a combination of vitamin B_12_ and folate. The sample size per study ranged from 12 to 807 participants. Most studies (17) were conducted in the USA, 4 each in the UK and India, and a small number of studies in 13 other countries. Gale 1990 [25] did not report where the study was conducted. Ten studies reported on or provided subgroup data for ELBW infants, and 5 reported on EP infants. Half the reports were published before the year 2000, and 40% in the last 15 years (Tables 1–6).

**Table 1:** Methodological details of included studies on vitamin A supplementation.

Study ID *Attached reports*	Location	Participants	Exposure	Comparator	Outcomes
Ambalavanan 2003 [26]	USA	BW 401–1000 g	10000 IU (Aquasol A) Intramuscular 3x per week 4 weeks	Standard care: 5000 IU (Aquasol A) Intramuscular 3x per week	ROP ROP, severe NEC Sepsis, proven Death
			15000 IU (Aquasol A) Intramuscular 1x per week 4 weeks		
Bental 1994 [23] Bental 1990 [27]	South Africa	BW 500–1000 g	4000 IU (Arovit) Intramuscular 3 x per week 4 weeks	Standard care All infants, if indicated: oral multivitamin (1500–3000 IU vitamin A)	IVH IVH, severe NEC Sepsis, proven BPD Death
Calisici 2014 [28] (abstract only)	Turkey	≤ 32 weeks GA at birth BW ≤ 1250 g	30000 IU.kg^-1^ Enteral Weekly 6 weeks	*Not reported*	*ROP (narrative)* *IVH, severe (narrative)* *Sepsis (narrative)* *BPD (narrative)* *Death (narrative)*
Giridhar 2020 [29]	India	BW 750–1250 g	5000 IU intramuscular (Aquasol A) or 10000 IU enteral (retinol palmitate)^1^ Alternate days 4 weeks	Placebo (saline)	IVH Sepsis, suspected BPD Death
Kiatchoosakun 2014 [30]	Thailand	BW < 1500 g	5000 IU (Chochola A) Intramuscular 3 x per week 4 weeks	Sham injections	ROP IVH, severe NEC Sepsis, proven BPD Death
Koo 1995 [31]	USA (2 sites)	BW ≤ 1500	High, medium, and low enriched formula^2^ Enteral Daily Until discharge	*NA*	*Growth (not included in the meta-analysis)* ^*3*^
Mactier 2012 [32] Hamilton 2010 [33] Mactier 2011 [34] McCulloch 2012 [35] EUCTR2005-003402-29-GB [36] NCT00417404 [37]	UK (2 sites)	< 32 weeks GA at birth BW ≤ 1500	10000 IU (Aqasol A) Intramuscular 3 x per week Until oral feeds are established (day 14) or for max. 12 doses	Sham injections	ROP ROP, severe IVH, severe BPD Death
Meyer 2024 [38] Meyer 2017 [39] Meyer 2016 [40] Meyer 2014 [41] DRKS00006541 [42] EUCTR2013-001998-24-DE [43]	Germany (27 sites) Austria (2 sites)	≤ 32 weeks GA at birth BW < 1000 g	5000 IU.kg^-1^ Enteral Daily 4 weeks	Placebo (peanut oil drops) All infants: basic supplementation of 1000 IU.kg^-1^.d^-1^ vitamin A with IVs or enteral	ROP ROP, severe, IVH IVH, severe NEC BPD Sepsis, proven Growth^4^ Death
Papagaroufalis 1991 [44]	Greece	VLBW	5000 IU initial dose, then 3750 IU (Arovit) every second day Intramuscular 8 injections (15 days)	Placebo (saline injections)	IVH IVH, severe Death
Papagaroufalis 1992 [45]	Greece	< 31 weeks GA at birth BW < 1500 g	Varied doses^5^ (Arovit) Intramuscular Varied regimen^5^ 6 weeks	Placebo	ROP ROP, severe
Pearson 1992 (33)	USA (2 sites)	BW 700–1100 g	2000 IU (Aquasol A) Intramuscular Every other day 4 weeks	Placebo (saline) or sham injections depending on study site	ROP *NEC (narrative)* *Sepsis (narrative)* BPD Death
Rakshasbhuvankar 2021 [46] Rakshasbhuvankar 2017 [47]	Australia	< 28 weeks GA at birth	5000 IU (Bio-Logical Vitamin A Solution) Enteral Daily Until 34 weeks PMA	Placebo (saline and coloring agent)	ROP, severe NEC Sepsis, suspected BPD Growth^6^ Death
Ravishankar 2003 [48]	USA	< 32 weeks GA at birth BW 500–1500 g	Weight-dependent dose (Aquasol A)^7^ Intramuscular On study days 1, 3, and 7	*Not reported*	IVH, severe NEC BPD Death
Schwarz 1997 [49]	USA	BW 750–1500 g	2500 IU (Aquasol A) Enteral Daily 3 weeks	Standard care All infants <1 kg: 690 IU.d^-1^ vitamin A in PN; > 1 kg: 1518 IU.d^-1^ vitamin A in PN; 1400−1500 IU.d^-1^ in enteral feeds (formula and/or fortifier)	ROP Growth^8^
Shenai 1987 [50]	USA	BW 700–1300 g 26-30 weeks GA at birth	2000 IU (Aquasol A) Intramuscular Every other day 4 weeks	Placebo (saline injections)	ROP BPD Death
Sun 2020 [51]	China (3 sites)	< 28 weeks GA at birth	1500 IU (Qingdao Double Whale Pharmaceutical) Enteral Daily 4 weeks	Placebo (soybean oil)	ROP IVH, severe NEC Sepsis, proven BPD Death
Tyson 1999 [52] Kennedy 1997 [53] Tyson 2000 [54] Ambalavanan 2005 [55] NCT01203488 [56]	USA (10 sites)	BW 401–1000 g Mechanical ventilation or supplemental oxygen at 24 hrs of age	5000 IU (Aquasol A) Intramuscular 3 x per week 4 weeks	Sham injections	Neurodevelopment Cognitive impairment Motor impairment Blindness Deafness Cerebral palsy IVH IVH, severe BPD NEC Sepsis, proven Death
Wardle 2001 [57]	UK	BW < 1000 g	5000 IU Enteral Daily 4 weeks	Placebo (visually identical solution)	ROP, severe Pulmonary haemorrhage NEC BPD Death

BPD, bronchopulmonary dysplasia; BW, birth weight; GA, gestational age; IVH, intraventricular haemorrhage; IU, international units; NEC, necrotising enterocolitis; ROP, retinopathy of prematurity; UK, United Kingdom; USA, United States of America; VLBW, very low birth weight

^1^Intramuscular administration until enteral fluid intake exceeded 50% or daily requirements, then switched to enteral.

^2^The low vitamin A enriched formula contained 820 IU per litre, the medium 1640 IU per litre, and the high 2900 IU per litre.

^3^This study was not included in the meta-analysis, because there was overlap in the cohort with another study on vitamin A supplementation. Growth was measured as weight gain (g.d^-1^), length gain (cm.d^-1^), or head circumference gain (cm.d^-1^) from birth to discharge or weight of 2000 g.

^4^Weight, head circumference, and length from birth to 36 weeks postmenstrual age.

^5^5000 IU initial dose, then 3750 IU every other day for two weeks, then 5000 IU weekly for 4 weeks.

^6^Weight gain from birth to discharge.

^7^1500 IU for 500–750 g, 2000 IU for 750–1000 g, 2500 IU for 1000–1250 g, 3000 IU for 1250–1500 g.

^8^Weight at study conclusion relative to birth weight (%).

**Table 2:** Methodological details of included studies on vitamin D supplementation.

Study ID *Attached reports*	Location	Participants	Exposure	Comparator	Outcomes
Anderson-Berry 2017 [58] Hanson 2014 [59]	USA	< 32 weeks GA at birth	800 IU (D3) Enteral Daily Duration of NICU hospitalisation	400 IU All infants: additional baseline vitamin D in IV nutrition	BPD *Growth (narrative)*
Evans 1989 [60]	Canada	BW < 1500 g	2000 IU (D2) Enteral Daily 6 weeks	400 IU	Growth (g.d^-1^) Death
Fort 2016 [61] Salas 2018 [62] Craig 2014 [63] Aristizabal 2023 [64]	USA	23 to < 28 weeks GA at birth	200 IU (D-Vi-Sol) Enteral (1 feed per day) Daily Until day 28 after birth	Standard care All infants: additional 200 IU vitamin D in IV or enteral nutrition solutions	Neurodevelopment Cognitive impairment Language impairment ROP ROP, severe IVH IVH, severe BPD NEC Sepsis, proven Growth Death
			200 IU (D-Vi-Sol) Enteral (200 IU for 4 feeds a day) Daily Until day 28 after birth		
Ge 2022 [65]	China	< 32 weeks GA at birth BW < 1500 g	800 IU (Qingdao Shuangjing Pharmaceutical) Enteral Daily 4 weeks	Standard care	BPD
Koo 1995 [66]	USA (2 sites)	BW ≤ 1500	High, medium, and low enriched formula^1^ Enteral Daily Until discharge	*NA*	*Growth (not included in the meta-analysis)* ^*2*^

BPD, bronchopulmonary dysplasia; BW, birth weight; GA, gestational age; IV, intravenous; IVH, intraventricular haemorrhage; IU, international units; NEC, necrotising enterocolitis; ROP, retinopathy of prematurity; USA, United States of America

^1^The low vitamin D enriched formula contained 177 IU of vitamin D per MJ, the medium 353 IU/MJ, the high 783 IU/MJ.

^2^This study was not included in the meta-analysis, because there was overlap in the cohort with another study on vitamin A supplementation. Growth was measured as weight gain (g.d^-1^), length gain (cm.d^-1^), or head circumference gain (cm.d^-1^) from birth to discharge or weight of 2000 g.

**Table 3:** Methodological details of included studies on vitamin E supplementation.

Study ID *Attached reports*	Location	Participants	Exposure	Comparator	Outcomes
Barekatain 2018 [67] Barekatain 2017 [68] IRCT2015042521910N2 [69]	Iran	≤ 30 weeks GA at birth	10 IU Enteral Daily 3 days	Placebo (distilled water)	IVH IVH, severe NEC Sepsis, proven Sepsis, suspected Death
Bell 2013 [70]	USA (14 sites)	< 27 weeks GA at birth BW < 1000 g	50 IU.kg^-1^ (Aquasol E) Enteral Single dose within 4 hrs of birth	Placebo (sterile water)	*IVH, severe (narrative)* *NEC (narrative)* *Sepsis* *Death (narrative)*
Chiswick 1983 [71]	UK	< 32 weeks GA at birth BW < 1751 g	20 mg.kg^-1^.d^-1^ (Ephynal) Intramuscular Daily 3 days	*Not reported*	IVH Death
Finer 1982 [72] Finer 1983 [73] Finer 1983 [74] Finer 1981 [75]	Canada	BW 750–1500 g	Varied doses^1^ (Ephynal) Intramuscular and enteral Varied regimen^1^ Until discharge	*Not reported*	ROP ROP, severe BPD Death
Fish 1990 [76]	USA	BW ≤ 1000 g	Varied doses^2^ (Ephynal) Intramuscular Varied regimen^2^ 6 days	Placebo (drug vehicle)	IVH IVH, severe NEC Sepsis, proven Death
Gale 1990 [25]	*NI*	BW < 2000 g (subgroup data for ≤ 1000 g provided and used here)	5 mg.kg^-1^ (Viprimol) Intra-arterial One continuous 8 infusion within 24 hrs after birth	Placebo (saline)	IVH
Hittner 1981 [77] Hittner 1982 [78] Hittner 1984 [22]	USA	BW ≤ 1500 g	100 mg.kg^-1^ (D/L-α-tocopherol in PG-PS80^3^) Enteral Daily Until discharge	5 mg.kg^-1^.d^-1^	ROP ROP, severe IVH IVH, severe NEC Sepsis, proven BPD Death
Johnson 1989 [79] Johnson 1985 [80] Schaffer 1988 [24]	USA (3 sites)	BW < 2000 g (subgroup data for < 1000 g provided and used here)	Varied doses^4^ (Ephynal) Intravenous, intramuscular, or enteral^4^ At least 8 weeks	Placebo (vehicle)	ROP IVH NEC Sepsis, proven BPD Death
Pathak 2003 [81]	USA (3 sites)	≤ 32 weeks GA at birth BW ≤ 1250 g	50 IU Enteral Daily 8 weeks	Placebo (vehicle)	NEC Sepsis, proven Anaemia^5^ Growth^6^ Death
Romero-Maldonado 2021 [82] NCT03274596 [83]	Mexico	VLBW	25 IU (Eternal) Enteral 2 x 12.5 IU daily Until 28 days after birth	Placebo (distillate water)	ROP IVH NEC Sepsis, suspected BPD Death
Sinha 1987 [84]	UK	≤ 32 weeks GA at birth	20 mg.kg^-1^ (Ephynal) Intramuscular Daily 48 hrs (3 doses)	Control (no treatment)	IVH NEC *Sepsis (narrative)* *Death (narrative)*
Speer 1984 [85] Chiswick 1991 [86]	USA	BW < 1500 g	Varied doses^2^ (Ephynal) Intramuscular Varied regimen^2^ 6 days	Placebo	ROP IVH IVH, severe NEC Sepsis, proven BPD Death
Tripathi 2011 [87]	India	BW < 1500 g < 37 weeks GA at birth	5 IU (Evion Pediatric) Enteral Daily 3 weeks	Control (no treatment)	Growth^7^
Watts 1991 [88] Watts 1981 [89]	Canada	BW < 1500 g	25 IU (Ephynal) Enteral Daily 6 weeks	Placebo (drug vehicle)	*NEC (narrative)* *Sepsis (narrative)* BPD Death

BPD, bronchopulmonary dysplasia; BW, birth weight; GA, gestational age; IVH, intraventricular haemorrhage; IU, international units; NEC, necrotising enterocolitis; ROP, retinopathy of prematurity; UK, United Kingdom; USA, United States of America; VLBW, very low birth weight

^1^25 mg within 12 hours of birth, 25 mg 12 hours later, followed by 20 mg IM daily for the next 14 days, then 20 mg IM every 3 days for 5 more doses, and after than 100 IU daily enteral, if tolerated, and if not, continued 20 mg IM every 3 days until enteral was tolerated.

^2^15, 10, 10, and 10 mg.kg^-1^ on days after birth 1, 2, 4, and 6, respectively.

^3^propylene glycol-polysorbate 80

^4^Dose dependent on vitamin E status, adjusted at least twice a week, to maintain target serum vitamin E concentration of 5 mg.dL^-1^. Given intravenously with fluids, as injections, or with enteral feeds once established.

^5^Mean number of transfusions per infant.

^6^Weight gain from birth to discharge.

^7^Weight gain from birth to conclusion of intervention.

**Table 4:** Methodological details of included studies on vitamin K supplementation.

Study ID *Attached reports*	Location	Participants	Exposure	Comparator	Outcomes
Hunnali 2023 [90] CTRI/2022/02/040396 [91]	India	≤ 32 weeks GA at birth BW ≤ 1500 g	0.5 mg (Kendadione) Intramuscular Once within 1 hr of birth	Standard care (1 mg once within 1 hr of birth)	IVH Pulmonary haemorrhage NEC Death
			0.3 mg (Kendadione) Intramuscular Once within 1 hr of birth		
Sethi 2019 [92] Data provided on request	India	BW ≤ 1500 g On antibiotic treatment for sepsis	1 mg (Kenadione or Reokay) Intramuscular Once on 7^th^ day of antibiotic treatment	Standard care (no treatment) *All infants received IM vitamin K at birth (≥1000 g 1 mg and <1000 g 0.5 mg)*	Clinical bleeding

BW, birth weight; GA, gestational age; IVH, intraventricular haemorrhage; NEC, necrotising enterocolitis

**Table 5:** Methodological details of included studies on vitamin C supplementation.

Study ID *Attached reports*	Location	Participants	Exposure	Comparator	Outcomes
Bass 1998 [93]	USA	BW < 1500 g	100 mg.kg^-1^ Arterial catheter Daily 7 days	Placebo (saline) *All infants: 10−30 mg.kg*^*-1*^*.d*^*-1*^ *in standard IV solution*	IVH IVH, severe BPD Sepsis, proven Anaemia^1^ Death
Darlow 2005 [94]	New Zealand	BW < 1500 g *or* < 32 weeks GA at birth	20 mg.kg^-1^ (Soluvit N, Vitdadol C) Parenteral or enteral Daily 28 days (from day 1)	Standard care parenteral feeds and placebo (sterile water) enteral feeds *All infants: 10 mg.kg*^*-1*^*.d*^*-1*^ *in standard IV solution*	ROP NEC BPD Death
			30 mg.kg^-1^ (Soluvit N, Vitdadol C) Parenteral or enteral Daily 18 days (from day 11)		
Doyle 1997 [95]	USA and Canada (multiple sites)	BW 1000–1500 g	50 mg (Ce-Vi-Sol) Enteral Daily 2 weeks	Placebo (drug vehicle) *All infants: 10 mg.kg*^*-1*^*.d*^*-1*^ *in PN solution, various doses in vitamin C enriched formula, fortifier and oral drops*	Anaemia^2^ Growth (g.d^-1^)

BPD, bronchopulmonary dysplasia; BW, birth weight; GA, gestational age; IVH, intraventricular haemorrhage; IU, international units; NEC, necrotising enterocolitis; ROP, retinopathy of prematurity; USA, United States of America

^1^n of transfusions per infant.

^2^n of infants who required transfusions.

**Table 6:** Methodological details of included studies on vitamin B_12_ and folic acid supplementation.

Study ID *Attached reports*	Location	Participants	Exposure	Comparator	Outcomes
Haiden 2006 [96]	Austria	BW 801–1300 g ≤ 32 weeks GA at birth	B_12_: 3 µg.kg^-1^.d^-1^ intravenously; 21 µg.kg^-1^.week^-1^ subcutaneously Folic acid: 100 µg.kg^-1^.d^-1^ enteral^1^ To 40 weeks CGA or discharge	Folic acid only 60 µg.kg^-1^.d^-1^ All infants: erythropoietin, iron, and vitamin E	NEC Anaemia^2^ Death

BW, birth weight; GA, gestational age; NEC, necrotising enterocolitis

^1^Vitamin B_12_ was added to PN daily until enteral feeds were established (≥60 mL.kg^-1^) and then given subcutaneously weekly. Folic acid was given orally as soon as enteral feeds were established or on day 15 after birth if feeds were not established.

^2^n of infants who required transfusions.

We identified 17 studies awaiting classification and 8 ongoing studies (S1 Table). Authors of studies that included a wider preterm population, i.e., not only VP and VLBW infants, were contacted, and subgroup data for VP and VLBW infants were requested. Authors of studies that continued supplementation after hospital discharge were contacted to provide outcome data at discharge. Authors of clinical trial registrations without a locatable publication of results were contacted for updates on the trial progress. If no responses were received, the studies were labelled as awaiting classification.

### Risk of bias assessment

**Fig 2:**
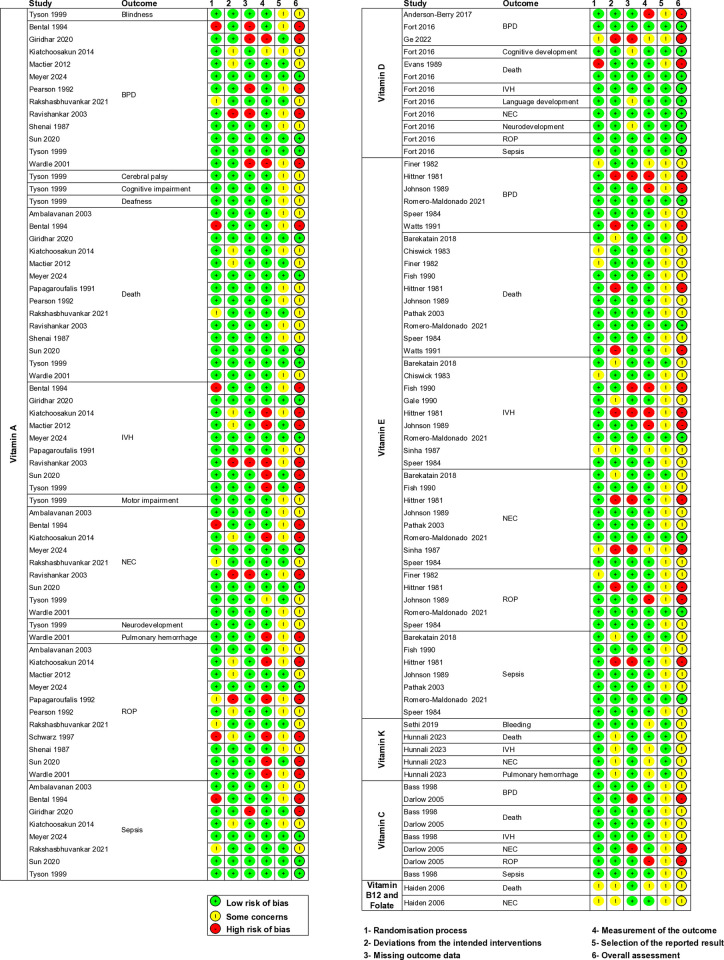
Risk of bias assessment. BPD, bronchopulmonary dysplasia; IVH, intraventricular haemorrhage; NEC, necrotizing enterocolitis; ROP, retinopathy of prematurity.

The risk of bias was assessed for 190 outcomes included in the meta-analysis and qualitative analysis (S2 Table). The risk of bias assessment for GRADE outcomes is summarised in Fig 2. The included studies were of variable methodological quality and rigour. Overall, 70 (37%) outcomes were found to be at high risk of bias, 84 (44%) at unclear risk of bias, and 36 (19%) at low risk of bias. Common reasons for the high or unclear risk of bias were lack of masking of staff, missing outcome data, inadequate description of data collection methods (e.g., time when an outcome was evaluated), and lack of published protocol or trial registration. If the study subjects were unmasked, but an adequate placebo was implemented (e.g., saline injections), the study was assessed as if the infants were masked. If no adequate placebo (e.g., sham injections) was implemented, the study was assessed as unmasked. All studies were assessed according to the intention-to-treat principle.

### Primary outcome

**Fig 3:**
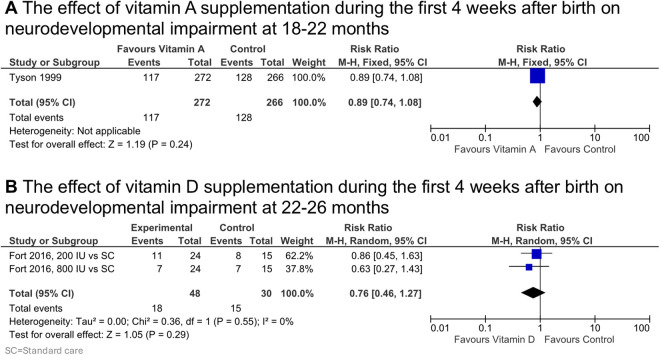
Primary outcome: effect of vitamin A or vitamin D supplementation during the first 4 weeks after birth on neurodevelopmental impairment at 2 years. CI, confidence interval; M-H, Mantel-Haenszel method.

Two studies reported data for the primary outcome. Ambalavanan 2005 [55] (follow-up report of Tyson 1999 [52]) reported neurodevelopment at 18–22 months after vitamin A supplementation. Tyson 1999 investigated the effects of 5000 IU vitamin A given intramuscularly 3 times per week for the first 4 weeks after birth in ELBW infants. They reported neurodevelopment as a composite outcome of motor impairment (consisting of Bayley II Motor Development Index <70 or Psychomotor Development Index <70), cerebral palsy, complete blindness, or deafness (hearing aids in both ears). They found no effect of neonatal vitamin A supplementation on the composite outcome (RR 0.89 [0.74, 1.08], p = 0.24, n = 538, very low certainty of evidence) nor on the individual components (Cognitive impairment: RR 0.83 [0.66, 1.04], p = 0.11; Motor impairment: RR 0.83 [0.63, 1.10], p = 0.19; Blindness: RR 4.98 [0.24, 103.33], p = 0.30; Deafness RR 1.24 [0.34, 4.58],: p = 0.32; Cerebral palsy: RR 0.83 [0.57, 1.20], p = 0.22) at 18–22 months(Fig 3 and S1 Fig).

Salas 2018 [62] (follow-up report of Fort 2016 [61]) reported neurodevelopment at 22–26 months after vitamin D supplementation. Vitamin D was given in 3 different groups (no supplementation, daily 200 IU or daily 800 IU) through an orogastric tube during the first 4 weeks after birth. All infants received 200 IU daily in parenteral nutrition. They reported neurodevelopment as a composite outcome of Bayley III cognitive composite score <85, moderate or severe cerebral, hearing impairment, or visual impairment in both eyes and found no effect of neonatal vitamin D supplementation on the composite outcome (RR 0.76 [0.46, 1.27], p = 0.29, n = 78, 1 study, very low certainty of evidence) nor the component outcomes separately (Cognitive impairment: RR 0.84 [0.45, 1.58], p = 0.60; Language impairment: RR 0.80 [0.53, 1.21], p = 0.29), at 22–26 months (Fig 3 and S1 Fig).

### Secondary outcomes

Studies investigating vitamins A, D, E, K, C, and a combination of vitamin B_12_ and folate reported data on the secondary outcomes. Data were available for all predefined outcomes. The results from the meta-analysis and the corresponding certainty of evidence assessments are summarised in Tables 7–12.

**Table 7:** Summary of findings – vitamin A.

Outcome	n of studies	n of patients		Effect				Certainty
		Vitamin A supplementation	no/lower dosage supplementation	Relative effect (95% CI)	Absolute effect (95% CI)	Risk difference (95% CI)	p-value for the effect estimate	
**Primary outcome at 18–22 months**								
**Neurodevelopmental impairment**	1	117/272 (43.0%)	128/266 (48.1%)	**RR 0.89** (0.74 to 1.08)	**53 fewer per 1,000** (from 125 fewer to 38 more)	**−0.05** (−0.14 to 0.03)	0.24	**⨁○○○** Very low^a, b^
**Cognitive impairment**	1	87/271 (32.1%)	104/268 (38.8%)	**RR 0.83** (0.66 to 1.04)	**66 fewer per 1,000** (from 132 fewer to 16 more)	**−0.07** (−0.15 to 0.01)	0.11	**⨁○○○** Very low^a, b^
**Motor impairment**	1	66/268 (24.6%)	79/266 (29.7%)	**RR 0.83** (0.63 to 1.10)	**50 fewer per 1,000** (from 110 fewer to 30 more)	**−0.05** (−0.13 to 0.02)	0.19	**⨁○○○** Very low^a, b^
**Blindness in both eyes**	1	2/289 (0.7%)	0/288 (0.0%)	**RR 4.98** (0.24 to 103.33)	**0 fewer per 1,000** (from 0 fewer to 0 fewer)	**0.01** (−0.00 to 0.02)	0.30	**⨁○○○** Very low^a, b^
**Deafness**	1	5/290 (1.7%)	4/288 (1.4%)	**RR 1.24** (0.34 to 4.58)	**3 more per 1,000** (from 9 fewer to 50 more)	**0.00** (−0.02 to 0.02)	0.75	**⨁○○○** Very low^a, b^
**Cerebral Palsy**	1	43/287 (15.0%)	51/283 (18.0%)	**RR 0.83** (0.57 to 1.20)	**31 fewer per 1,000** (from 77 fewer to 36 more)	**−0.03** (−0.09 to 0.03)	0.33	**⨁○○○** Very low^a, b^
**Secondary outcomes during initial hospital stay after birth**								
**Retinopathy of prematurity, all stages**	11	351/1004 (35.0%)	420/985 (42.6%)	**RR 0.77** (0.61 to 0.98)	**98 fewer per 1.000** (from 166 fewer to 9 fewer)	**−0.10** (−0.17 to −0.02)	0.03	**⨁⨁○○** Low^c, **d**^
**Retinopathy of prematurity, severe**	6	81/779 (10.4%)	92/766 (12.0%)	**RR 0.77** (0.46 to 1.28)	**28 fewer per 1.000** (from 65 fewer to 34 more)	**−0.05** (−0.13 to 0.02)	0.31	**⨁○○○** Very low ^c, e^
**Intraventricular haemorrhage, all grades**	9	330/1205 (27.4%)	342/1214 (28.2%)	**RR 0.96** (0.80 to 1.16)	**11 fewer per 1.000** (from 56 fewer to 45 more)	**−0.01** (−0.05 to 0.03	0.69	**⨁⨁○○** Low^c, e^
**Intraventricular haemorrhage, severe**	8	133/1144 (11.6%)	150/1155 (13.0%)	**RR 0.85** (0.64 to 1.14)	**19 fewer per 1.000** (from 47 fewer to 18 more)	**−0.02** (−0.06 to 0.02)	0.29	**⨁○○○** Very low^c, e, f^
**Pulmonary haemorrhage**	1	5/77 (6.5%)	11/77 (14.3%)	**RR 0.45** (0.17 to 1.25)	**79 fewer per 1.000** (from 119 fewer to 36 more)	**−0.08** (−0.17 to 0.02)	0.13	**⨁○○○** Very low^a. b, g^
**Bronchopulmonary dysplasia**	12	697/1309 (53.2%)	771/1309 (58.9%)	**RR 0.89** (0.78 to 1.01)	**65 fewer per 1.000** (from 130 fewer to 6 more)	**−0.06** (−0.10 to −0.01)	0.06	**⨁⨁⨁○** Moderate^c^
**Necrotising enterocolitis**	9	119/1330 (8.9%)	130/1296 (10.0%)	**RR 0.87** (0.69 to 1.10)	**13 fewer per 1.000** (from 31 fewer to 10 more)	**−0.01** (−0.03 to 0.01)	0.26	**⨁⨁○○** Low^c, e^
**Sepsis, culture-proven**	6	255/1137 (22.4%)	262/1107 (23.7%)	**RR 0.88** (0.77 to 1.00)	**28 fewer per 1.000** (from 54 fewer to 0 fewer)	**−0.02** (−0.06 to 0.02)	0.05	**⨁⨁○○** Low^e,f^
**Sepsis, culture-proven or suspected**	8	308/1292 (23.8%)	320/1260 (25.4%)	**RR 0.88** (0.78 to 0.99)	**30 fewer per 1.000** (from 56 fewer to 3 fewer)	**−0.02** (−0.06 to 0.01)	0.04	**⨁⨁⨁○** Moderate^c^
**Death**	14	179/1504 (11.9%)	169/1467 (11.5%)	**RR 0.97** (0.80 to 1.18)	**3 fewer per 1.000** (from 23 fewer to 21 more)	**0.00** (−0.02 to 0.02)	0.76	**⨁⨁⨁○** Moderate^h^

CI, confidence interval; RR, risk ratio

***Explanations***

a. Downgraded two levels for indirectness because only one trial contributed to the outcome.

b. Downgraded two levels for imprecision because optimal sample size is not met, and 95% CI overlaps no effect.

c. Downgraded one level for risk of bias because the proportion of information from studies at high risk of bias is sufficient to affect the interpretation of results. There is a crucial limitation for one criterion, or some limitations for multiple criteria, sufficient to lower confidence in the estimate of effect.

d. I^2^ statistic shows the proportion of the variation in estimates due to differences between the trials is > 50%.

e. Downgraded one level for imprecision because 95% CI overlaps no effect.

f. Downgraded one level for indirectness because the mode of administration of intervention in the included trials for this outcome does not represent the generally included trials.

g. Downgraded two levels for risk of bias because the proportion of information from studies at high risk of bias is sufficient to affect the interpretation of results. There is a crucial limitation for one or more criteria sufficient to substantially lower confidence in the estimate of effect.

h. Downgraded one level for imprecision because optimal sample size is not met.

**Table 8:** Summary of findings – vitamin D.

Outcome	n of studies	n of patients		Effect				Certainty
		Vitamin D supplementation	no/lower dosage supplementation	Relative effect (95% CI)	Absolute effect (95% CI)	Risk difference (95% CI)	p-value for the effect estimate	
**Primary outcome at 22–26 months**								
Neurodevelopmental impairment	1	18/48 (37.5%)	15/30 (50.0%)	**RR 0.76** (0.46 to 1.27)	**120 fewer per 1,000** (270 fewer to 135 more)	**−0.13** (−0.35 to 0.10)	0.29	**⨁○○○** Very low^a,b^
Cognitive impairment	1	14/42 (33.3%)	11/28 (39.3%)	**RR 0.84** (0.45 to 1.58)	**63 fewer per 1,000** (216 fewer to 228 more)	**−0.06** (−0.29 to 0.17)	0.60	**⨁○○○** Very low^a,b^
Language impairment	1	21/41 (51.2%)	18/28 (64.3%)	**RR 0.80** (0.53 to 1.20)	**129 fewer per 1,000** (302 fewer to 129 more)	**−0.13** (−0.36 to 0.10)	0.29	**⨁○○○** Very low^a,b^
**Secondary outcomes during initial hospital stay after birth**								
Retinopathy of prematurity, all stages	1	25/64 (39.1%)	18/36 (50.0%)	**RR 0.79** (0.47 to 1.33)	**105 fewer per 1,000** (265 fewer to 165 more)	**−0.11** (−0.31 to 0.09)	0.38	**⨁○○○** Very low^a,b^
Retinopathy of prematurity, severe	1	4/64 (6.3%)	3/36 (8.3%)	**RR 0.75** (0.17 to 3.22)	**21 fewer per 1,000** (69 fewer to 185 more)	**−0.01** (−0.12 to 0.09)	0.70	**⨁○○○** Very low^a,b^
Intraventricular haemorrhage, all grades	1	45/64 (70.3%)	24/36 (66.7%)	**RR 1.05** (0.73 to 1.51)	**33 more per 1,000** (180 fewer to 340 more)	**0.04** (−0.22 to 0.29)	0.79	**⨁○○○** Very low^a,b^
Severe intraventricular haemorrhage, grade 3 and 4	1	15/64 (23.4%)	4/36 (11.1%)	**RR 2.04** (0.72 to 5.75)	**116 more per 1,000** (31 fewer to 528 more)	**0.11** (−0.03 to 0.26)	0.18	**⨁○○○** Very low^a,b^
Bronchopulmonary dysplasia/chronic lung disease	3	34/137 (24.8%)	43/107 (40.2%)	**RR 0.58** (0.41 to 0.83)	**169 fewer per 1,000** (from 237 fewer to 68 fewer)	**−0.18** (−0.29 to −0.07)	0.003	**⨁○○○** Very low^c,d^
Necrotising enterocolitis	1	5/64 (7.8%)	1/36 (2.8%)	**RR 1.63** (0.20 to 13.39)	**17 more per 1,000** (from 22 fewer to 344 more)	**0.05** (−0.09 to 0.18)	0.65	**⨁○○○** Very low^a,b^
Proven sepsis	1	24/64 (37.5%)	5/36 (13.9%)	**RR 0.68** (0.31 to 1.50)	**116 fewer per 1,000** (from 249 fewer to 81 more)	**−0.12** (−0.33 to 0.09)	0.34	**⨁○○○** Very low^a,b^
Growth, weight (g.d-1)	1	41	40	**MD 2.00** (−2.38 to 6.38)	**–**	**–**	0.37	NA
Mortality	2	10/109 (9.5%)	3/78 (3.9%)	**RR 1.43** (0.32 to 6.39)	**17 more per 1,000** (26 fewer to 207 more)	**0.05** (−0.08 to 0.17)	0.64	**⨁○○○** Very low^b,e^

CI, confidence interval; RR, risk ratio.

***Explanations***

a. Downgraded two levels for indirectness because only one trial contributed to the outcome.

b. Downgraded two levels for imprecision because optimal sample size is not met, and 95% CI overlaps no effect.

c. Downgraded two levels for risk of bias because the proportion of information from studies at high risk of bias is sufficient to affect the interpretation of results. There is a crucial limitation for one or more criteria sufficient to substantially lower confidence in the estimate of effect.

d. Downgraded one level for imprecision because optimal sample size is not met.

e. Downgraded one level for risk of bias because the proportion of information from studies at high risk of bias is sufficient to affect the interpretation of results. There is a crucial limitation for one criterion, or some limitations for multiple criteria, sufficient to lower confidence in the estimate of effect.

**Table 9:** Summary of findings – vitamin E.

Outcome	n of studies	n of patients		Effect				Certainty
		Vitamin E supplementation	no/lower dosage supplementation	Relative effect (95% CI)	Absolute effect (95% CI)	Risk difference (95% CI)	p-value for the effect estimate	
**Retinopathy of prematurity, all stages**	5	176/410 (42.9%)	200/412 (48.5%)	**RR 0.90** (0.73 to 1.11)	**49 fewer per 1.000** (from 131 fewer to 53 more)	**−0.07** (−0.13 to −0.01)	0.33	**⨁⨁○○** Low^a,b^
**Retinopathy of prematurity, severe**	2	0/98 (0.0%)	9/102 (8.8%)	**RR 0.10** (0.01 to 0.80)	**79 fewer per 1.000** (from 87 fewer to 18 more)	**−0.09** (−0.15 to −0.03)	0.03	**⨁○○○** Very low^a,c,d^
**Intraventricular haemorrhage, all grades**	9	141/659 (21.4%)	204/671 (30.4%)	**RR 0.70** (0.52 to 0.92)	**91 fewer per 1.000** (from 146 fewer to 24 fewer)	**−0.11** (−0.20 to −0.02)	0.01	**⨁⨁⨁○** Moderate^e^
**Intraventricular haemorrhage, severe**	4	24/220 (10.9%)	32/228 (14.0%)	**RR 0.79** (0.45 to 1.39)	**29 fewer per 1.000** (from 77 fewer to 55 more)	**−0.03** (−0.08 to 0.02)	0.42	**⨁○○○** Very low^b,f^
**Bronchopulmonary dysplasia**	6	153/623 (26.5%)	165/632 (27.5%)	**RR 0.94** (0.79 to 1.12)	**16 fewer per 1.000** (from 55 fewer to 31 more)	**−0.02** (−0.07 to 0.02)	0.48	**⨁○○○** Very low^f,g^
**Necrotising enterocolitis**	8	62/659 (9.4%)	48/672 (7.1%)	**RR 1.29** (0.90 to 1.85)	**21 more per 1.000** (from 7 fewer to 61 more)	**0.02** (−0.00 to 0.04)	0.17	**⨁⨁○○** Low^e,g^
**Sepsis, culture-proven**	6	87/509 (17.1%)	66/522 (12.6%)	**RR 1.34** (0.99 to 1.80)	**43 more per 1.000** (from 1 fewer to 101 more)	**0.04** (−0.00 to 0.08)	0.06	**⨁⨁○○** Low^e,g^
**Sepsis, culture-proven or suspected**	7	129/557 (23.2%)	104/564 (18.4%)	**RR 1.15** (0.91 to 1.47)	**28 more per 1.000** (from 17 fewer to 87 more)	**0.05** (0.00 to 0.09)	0.24	**⨁○○○** Very low^a,b^
**Anaemia, n of infusions per infant**	1	15	15	**MD −0.20** (−1.18 to 0.78)	**–**	**–**	0.96	NA
**Death**	10	132/793 (16.6%)	143/809 (17.7%)	**RR 0.98** (0.80 to 1.20)	**4 fewer per 1.000** (from 35 fewer to 35 more)	**−0.01** (−0.04 to 0.01)	0.81	**⨁⨁⨁○** Moderate^g^

CI, confidence interval; RR, risk ratio.

***Explanations***

a. Downgraded one level for risk of bias because most information is from studies at low or unclear risk of bias and potential limitations are likely to lower confidence in the estimate of effect.

b. Downgraded two levels for imprecision because optimal sample size is not met, and 95% CI overlaps no effect.

c. Downgraded one level for indirectness because the mode of administration of intervention in the included trials for this outcome does not represent the generally included trials.

d. Downgraded one level for imprecision because optimal sample size is not met.

e. Downgraded one level for risk of bias because the proportion of information from studies at high risk of bias is sufficient to affect the interpretation of results. There is a crucial limitation for one criterion, or some limitations for multiple criteria, sufficient to lower confidence in the estimate of effect.

f. Downgraded two levels for risk of bias because the proportion of information from studies at high risk of bias is sufficient to affect the interpretation of results. There is a crucial limitation for one or more criteria sufficient to substantially lower confidence in the estimate of effect.

g. Downgraded one level for imprecision because 95% CI overlaps no effect.

**Table 10:** Summary of findings – vitamin K.

Outcome	n of studies	n of patients		Effect				Certainty
		Vitamin K supplementation	no/lower dosage supplementation	Relative effect (95% CI)	Absolute effect (95% CI)	Risk difference (95% CI)	p-value for the effect estimate	
**Intraventricular haemorrhage, all grades**	1	0/24 (0.0%)	4/51 (7.8%)	**RR 0.41** (0.05 to 3.31)	**46 fewer per 1.000** (from 75 fewer to 181 more)	**−0.08** (−0.19 to 0.003)	0.40	**⨁○○○** Very low^a. b^
**Pulmonary haemorrhage**	1	0/24 (0.0%)	3/51 (5.9%)	**RR 0.52** (0.06 to 4.47)	**28 fewer per 1.000** (from 55 fewer to 204 more)	**−0.06** (−0.16 to 0.05)	0.55	**⨁○○○** Very low^a. b^
**Necrotising enterocolitis**	1	1/24 (4.2%)	5/51 (9.8%)	**RR 0.64** (0.11 to 3.87)	**35 fewer per 1.000** (from 87 fewer to 281 more)	**−0.06** (−0.19 to 0.06)	0.63	**⨁○○○** Very low^a. b^
**Clinical bleeding**	1	2/24 (8.3%)	1/11 (9.1%)	**RR 0.92** (0.09 to 9.07)	**7 fewer per 1,000** (from 83 fewer to 734 more)	**−0.01** (−0.21 to 0.20)	0.94	**⨁○○○** Very low^a. b^
**Death**	1	2/24 (8.3%)	3/51 (5.9%)	**RR 1.43** (0.25 to 8.20)	**25 more per 1.000** (from 44 fewer to 424 more)	**0.03** (−0.10 to 0.15)	0.69	**⨁○○○** Very low^a. b^

CI, confidence interval; RR, risk ratio.

***Explanations***

a. Downgraded two levels for indirectness because only one trial contributed to the outcome.

b. Downgraded two levels for imprecision because optimal sample size is not met, and 95% CI overlaps no effect.

**Table 11:** Summary of findings – vitamin C.

Outcome	n of studies	n of patients		Effect				Certainty
		Vitamin C supplementation	no/lower dosage supplementation	Relative effect (95% CI)	Absolute effect (95% CI)	Risk difference	p-value for the effect estimate	
**Retinopathy of prematurity, all stages**	1	21/55 (38.2%)	12/31 (38.7%)	**RR 0.99** (0.57 to 1.72)	**4 fewer per 1.000** (from 166 fewer to 279 more)	**−0.00** (−0.22 to 0.21)	0.97	**⨁○○○** Very low^a, b, c^
**Intraventricular haemorrhage, all grades**	1	8/23 (34.8%)	7/24 (29.2%)	**RR 1.19** (0.52 to 2.76)	**55 more per 1.000** (from 140 fewer to 513 more)	**0.06** (−0.21 to 0.32)	0.68	**⨁○○○** Very low^b, c^
**Intraventricular haemorrhage, severe**	1	0/23 (0.0%)	2/24 (8.3%)	**RR 0.21** (0.01 to 4.12)	**66 fewer per 1.000** (from 82 fewer to 260 more)	**−0.08** (−0.21 to 0.05)	0.30	**⨁○○○** Very low^b, c^
**Bronchopulmonary dysplasia**	2	24/97 (24.7%)	27/63 (42.9%)	**RR 0.59** (0.37 to 0.93)	**176 fewer per 1.000** (from 270 fewer to 30 fewer)	**−0.18** (−0.33 to −0.03)	0.02	**⨁○○○** Very low^d, e, f^
**Necrotising enterocolitis**	1	4/79 (5.1%)	1/40 (2.5%)	**RR 1.27** (0.17 to 9.25)	**14 more per 1.000** (from 19 fewer to 212 more)	**0.03** (−0.07 to 0.12)	0.82	**⨁○○○** Very low^a, b, c^
**Sepsis, culture-proven**	1	7/23 (30.4%)	11/24 (45.8%)	**RR 0.66** (0.31 to 1.41)	**156 fewer per 1.000** (from 316 fewer to 188 more)	**−0.15** (−0.43 to 0.12)	0.29	**⨁○○○** Very low^b, c^
**Anaemia, n of transfusions per infant**	1	25	26	**MD 0.10** (−1.36 to 1.56)	**–**	**–**	0.89	NA
**Anaemia, n of infants who required transfusions**	1	6/42 (14.3%)	3/39 (7.7%)	**RR 1.86** (0.50 to 6.92)	**66 more per 1.000** (38 fewer to 455 more)	**0.07** (−0.07 to 0.20)	0.36	NA
**Growth, weight (g.d**^**-1**^)	1	24	26	**MD 0.20** (−4.42 to 4.82)	**–**	**–**	0.93	NA
**Death**	2	4/64 (6.3%)	2/66 (3.0%)	**RR 1.99** (0.36 to 11.12)	**30 more per 1.000** (from 19 fewer to 307 more)	**0.03** (−0.04 to 0.10)	0.43	**⨁○○○** Very low^c, e^

CI, confidence interval; RR, risk ratio

***Explanations***

a. Downgraded two levels for risk of bias because the proportion of information from studies at high risk of bias is sufficient to affect the interpretation of results. There is a crucial limitation for one or more criteria sufficient to substantially lower confidence in the estimate of effect.

b. Downgraded two levels for indirectness because only one trial contributed to the outcome.

c. Downgraded two levels for imprecision because optimal sample size is not met, and 95% CI overlaps no effect.

d. Downgraded one level for risk of bias because the proportion of information from studies at high risk of bias is sufficient to affect the interpretation of results. There is a crucial limitation for one criterion, or some limitations for multiple criteria, sufficient to lower confidence in the estimate of effect.

e. Downgraded one level for indirectness because the mode of administration of intervention in the included trials for this outcome does not represent the generally included trials.

f. Downgraded one level for imprecision because optimal sample size is not met.

**Table 12:** Summary of findings – vitamin B_12_ and folic acid.

Outcome	n of studies	n of patients		Effect				Certainty
		Vitamin C supplementation	no/lower dosage supplementation	Relative effect (95% CI)	Absolute effect (95% CI)	Risk difference	p-value for the effect estimate	
**Necrotising enterocolitis**	1	1/31 (3.2%)	3/33 (9.1%)	**RR 0.35** (0.04 to 3.23)	**59 fewer per 1.000** (from 87 fewer to 203 more)	**−0.06** (−0.17 to 0.06)	0.36	**⨁○○○** Very low^a, b, c^
**Anaemia, n of infants who required transfusions**	1	7/31 (22.6%)	10/33 (30.3%)	**RR 0.75** (0.32 to 1.71)	**76 fewer per 1.000** (from 206 fewer to 215 more)	**−0.08** (−0.29 to 0.14)	0.48	**NA**
**Death**	1	3/31 (9.7%)	2/33 (6.1%)	**RR 1.60** (0.29 to 8.92)	**36 more per 1.000** (from 43 fewer to 480 more)	**0.04** (−0.10 to 0.17)	0.59	**⨁○○○** Very low^a, b, c^

CI, confidence interval; RR, risk ratio

***Explanations***

a. Downgraded one level for risk of bias because most information is from studies at low or unclear risk of bias and potential limitations are likely to lower confidence in the estimate of effect.

b. Downgraded two levels for indirectness because only one trial contributed to the outcome.

c. Downgraded two levels for imprecision because optimal sample size is not met and 95% CI overlaps no effect

**Fig 4:**
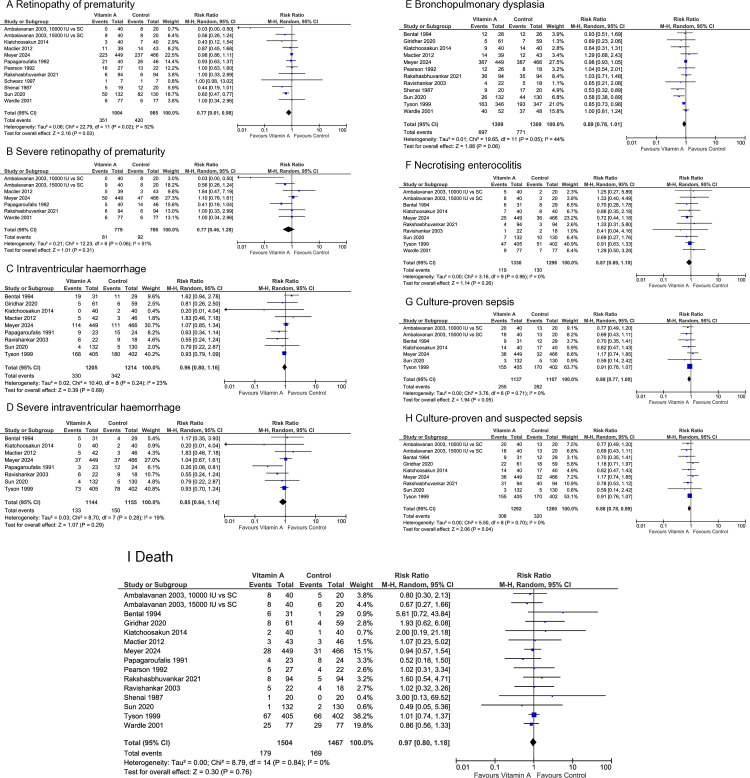
Effect of vitamin A supplementation on select secondary outcomes. CI, confidence interval; M-H, Mantel-Haenszel method.

The evidence suggests vitamin A supplementation reduces the risk of ROP (RR 0.77 [0.61, 0.98], p = 0.03, NNT = 14, n = 1989, 11 studies, low certainty of evidence) and proven and suspected sepsis (RR 0.88 [0.78, 0.99], p = 0.04, NNT = 65, n = 2552, 8 studies, moderate certainty of evidence) (Fig 4). The 16 studies that contributed data to the quantitative analysis investigated a wide variety of enteral and intramuscular administration doses, duration, and regimens. Studies were conducted in 9 different countries, and 44% investigated or provided subgroup data for extremely preterm or extremely low birth weight infants.

The evidence suggests that vitamin A supplementation may result in little to no difference in severe ROP, all grade IVH, pulmonary haemorrhage, BPD, culture-proven sepsis, NEC, and mortality (Fig 4 and S1 Fig).

**Fig 5:**
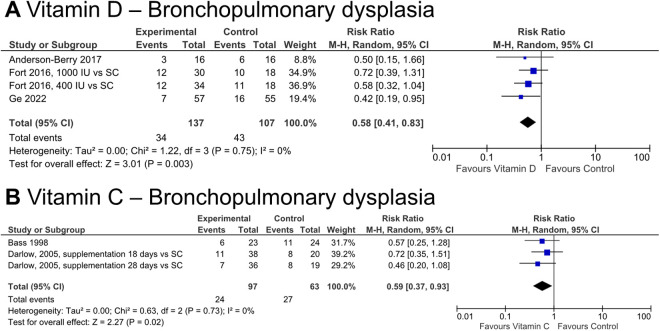
Effect of vitamin D and vitamin C supplementation on select secondary outcomes. CI, confidence interval; M-H, Mantel-Haenszel method.

Vitamin D may reduce the risk of BPD (RR 0.58 [0.41, 0.83], p = 0.003, NNT = 7, n = 244, 3 studies, very low certainty of evidence) (Fig 5). 4 studies contributed data for the quantitative analysis. All studies investigated daily enteral administration of 200–2000 IU vitamin D daily for at least 4 weeks. They were conducted in 3 different countries; one study investigated extremely preterm infants.

Vitamin D may have little to no effect on any stage or severe ROP, any grade or severe IVH, NEC, culture-proven sepsis, and mortality, but the evidence is very uncertain (S1 Fig).

**Fig 6:**
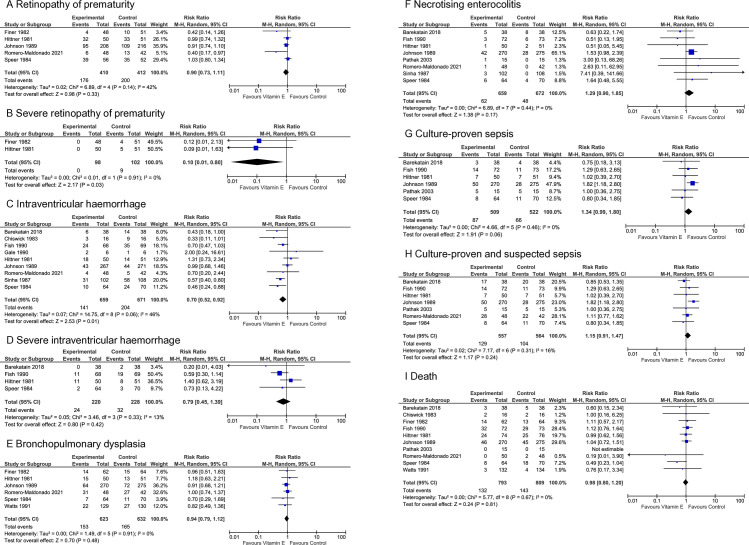
Effect of vitamin E supplementation on select secondary outcomes. CI, confidence interval; M-H, Mantel-Haenszel method.

The evidence suggests vitamin E supplementation reduces the risk of severe ROP (RR 0.10 [0.01, 0.80], p = 0.03, NNT = 12, n = 200, 2 studies, very low certainty of evidence) and any grade IVH (RR 0.70 [0.52, 0.92], p = 0.01, NNT = 12, n = 1330, 9 studies, moderate certainty of evidence) (Fig 6). Eleven studies contributed data for the quantitative analysis. They investigated a wide variety of enteral, intravenous, and intramuscular administration doses, duration, and regimens. Studies were conducted in 5 different countries; for one study, the location was not clear. About half of the studies investigated or provided subgroup data on extremely low birth weight infants.

The evidence suggests that vitamin E supplementation may result in little to no difference in any stage ROP, severe IVH, BPD, NEC, culture-proven or suspected sepsis, and mortality (Fig 6 and S1 Fig).

Two studies were included for vitamin K. Sethi 2019 [92] included 80 neonates of any gestational age, but authors were contacted and provided data for the 49 participants born < 33 weeks of gestation. All infants in the two trials received intramuscular vitamin K of varying dosages. The evidence is very uncertain on the effect of different doses of vitamin K on any grade IVH, pulmonary haemorrhage, NEC, mortality, and a compound outcome of any type of clinical bleed defined by the authors (S1 Fig).

Vitamin C may reduce the risk of BPD (RR 0.59 [0.37, 0.93], p = 0.02, NNT = 6, n = 160, 2 studies, very low certainty of evidence) (Fig 5). Three studies contributed data for the quantitative analysis, investigating daily enteral or intravenous administration of 30–100 mg.kg^-1^ for one to 4 weeks. Studies were conducted in 2 different countries, and no studies investigated only extremely low birth weight or extremely preterm infants.

Vitamin C may have little to no effect on any stage ROP, any grade or severe IVH, NEC, culture-proven sepsis, and mortality but the evidence is very uncertain (S1 Fig).

One study investigated a combination of vitamin B_12_ and folate supplementation. There were no differences between the supplemented and comparator groups in NEC, and mortality (S1 Fig).

Despite considerable heterogeneity in the methods of the included studies, especially regarding the mode and dose of vitamin supplementation, no statistical heterogeneity was detected in the data.

If meta-analysis of data was not possible, or studies provided data narratively, results were synthesised qualitatively in the S2 File. This includes studies reporting on the effects of supplementation on anaemia and growth outcomes.

### Sensitivity analysis

After exclusion of studies at high risk of bias for the outcome, there were no effects of vitamin A on ROP (RR 0.80 [0.58, 1.10], p = 0.17, n = 1393, n of studies removed = 5, n of studies remaining = 6). Similarly, vitamin E did not affect severe ROP (RR 0.12 [0.01, 2.13], p = 0.15, n = 99, n of studies removed = 1, n of studies remaining = 1), and vitamin C did not affect BPD (RR 0.57 [0.25, 1.28], p = 0.17, n = 47, n of studies removed = 1, n of studies remaining = 1).

### Subgroup analysis

Subgroup analyses could not be conducted for the primary outcome as only one study contributed data for vitamin A and D supplementation, respectively. Subgroup analyses by ethnicity were planned but could not be conducted due to a lack of data. Details on all conducted subgroup analyses can be found in S3 Table.

#### Birth weight and gestational age at birth.

Vitamin A supplementation showed no effect when only ELBW infants (up to 1081 infants) were included in the meta-analysis. Vitamin A supplementation decreased the risk of ROP for EP infants (RR 0.63 [0.49, 0.81], p < 0.001, n = 450, 2 studies).

For vitamin E, the subgroup analysis with only ELBW infants showed an increased risk of sepsis after supplementation (RR 1.76 [1.04, 2.99], p = 0.04, n = 291, 2 studies). The decreased risk of IVH persisted in ELBW infants (RR 0.70 [0.50, 0.98], p = 0.04, n = 349, 4 studies).

#### Mode of administration and regimen.

Only intramuscular, but not enteral vitamin A reduced the risk of BPD (RR 0.84 [0.74, 0.95], p = 0.006, n = 1033, 7 studies) and culture-proven sepsis (RR 0.85 [0.74, 0.98], p = 0.03, n = 1067, 4 studies) (S3 Table). In subgroup analyses of the administration regimen, alternate day intramuscular injections for 8 doses over 15 days reduced the risk of severe IVH, but only one study with 47 participants was included in the subgroup (RR 0.26 [0.08, 0.81], p = 0.02). Administration 3 times per week for 2–4 weeks was also shown to decrease the risk of BPD (RR 0.86 [0.75, 0.98], p = 0.03, n = 909, 4 studies, all intramuscular administration) but showed no significance for intramuscular administration on alternate days for 2–6 weeks, or for 3 doses in the first week after birth. There was no evidence of an interaction between different modes of administration and the effects of vitamins on outcomes.

Similarly, for vitamin E, there were no effects for studies investigating enteral or intravenous administration, but intramuscular administration reduced the risk of IVH (RR 0.58 [0.46, 0.73], p < 0.001, n = 513, 4 studies) (S3 Table). One study used a mixed administration approach, giving vitamin E intravenously while the infants were on intravenous fluids and enterally when enteral feeds were established, continuously monitoring serum vitamin E concentrations, and adjusting the dose to maintain a target concentration of 5 mg.dL^-1^. Intramuscular injections were used when enteral vitamin E did not successfully increase serum concentrations to the target. This approach increased the risk of culture-proven sepsis in the intervention group (RR 1.82 [1.18, 2.80], p = 0.007, n = 545, 1 study) (S3 Table). For the supplementation regimen, a regimen of intramuscular injections on days 1, 2, 4, and 6 after birth reduced the risk of IVH (RR 0.61 [0.41, 0.90], p = 0.01, n = 271, 2 studies) (S3 Table). Interaction tests demonstrated no subgroup differences between different modes of administration or administration regimens.

#### Country of birth.

86% of studies were conducted in high or upper-middle-income countries, 12% in lower-middle-income countries, and none in low-income countries. In one study, the country was not stated (Gale 1990 [25]).

Subgroup analysis was possible for vitamin A and vitamin E. Vitamin A supplementation reduced the risk of proven and suspected sepsis (RR 0.84 [0.74, 0.96], p = 0.01, n = 1517, 6 studies) in high and upper-middle-income countries. Vitamin E reduced the risk of IVH (RR 0.71 [0.53, 0.96], p = 0.03, n = 1242, 7 studies) and increased the risk of proven sepsis (RR 1.37 [1.01, 1.86], p = 0.04, n = 955, 5 studies) in high and upper-middle-income countries (S3 Table). Interaction tests demonstrated no subgroup differences between groups for either vitamin supplementation.

## Discussion

This systematic review included 43 studies investigating vitamin supplementation to reduce mortality and morbidities in 6096 VP and VLBW infants. We found very little evidence on the effects of vitamin supplementation during the neonatal period for this population on neurodevelopmental outcome at 2 years: only 2 studies reported follow-up at 2 years after neonatal vitamin A and vitamin D supplementation and found no effects.

However, supplementation with some vitamins may decrease the risk of neonatal morbidities during the initial hospital stay after birth. Vitamin A supplementation reduced the risk of ROP and sepsis, but not of BPD. These findings differ from a recent systematic review and meta-analysis on vitamin A supplementation in VLBW and VP infants, where vitamin A was shown to have beneficial effects on the risk of BPD at 36 weeks and any stage ROP but not sepsis [46]. Due to differences in inclusion criteria, two trials included in the previous review were not included in our review because supplementation was continued post-discharge, and two included in our review were not included in the former. In addition, one large trial (Meyer 2024 [38]) has been published since the previous review. Vitamin A has been shown to promote pulmonary epithelial cell growth, development, and differentiation and promote surfactant synthesis in the preterm infant [97,98]. Retinal, a vitamer of vitamin A, is a building block for photoreceptors in the human eye and is thus important for the development of vision [97]. Furthermore, vitamin A regulates mucosal immunity and immune response [99]. It has been suggested that immaturity of the preterm infant’s gastrointestinal tract leads to impaired intestinal absorption of fat-soluble vitamin formulations and that injections may be necessary to show any effect [100]. Our subgroup analyses showed that only intramuscular vitamin A reduced the risk of BPD and culture-proven sepsis, while there were no effects in a subgroup of enteral supplementation.

Even though vitamin D supplementation was shown to reduce the risk of BPD, only 3 studies and 244 infants were included. Additionally, 2 of the 3 studies were at high risk of bias, leading to the assessment that the evidence for this finding is very uncertain. Vitamin D is crucial for regulating calcium-phosphate metabolism, provided the intake of these minerals is adequate [101]. Vitamin D has recently been associated with immunity and inflammation regulation, binding to vitamin D receptor on cells of the innate and adaptive immune system [102–104]. BPD is at least partially an inflammatory disease, so modulation of the immune response through vitamin D supplementation is feasible and warrants further research.

Vitamin E reduced the risk of severe ROP, but only 2 studies were included with some concerns or high risk of bias and very low certainty of evidence. However, vitamin E was also shown to reduce the risk of any grade IVH with moderate certainty of evidence. Vitamin E has a strong antioxidative function, neutralising free radicals and protecting DNA, proteins, and lipids from oxidative damage. Oxidative stress and increased inflammation are prevalent in preterm infants and are contributing factors to the development of several preterm complications [105]. Meanwhile, excess intravenous vitamin E supplementation may have adverse effects, potentially increasing the risk of sepsis and haemorrhage [106]. We found that sepsis was slightly increased by vitamin E, with low certainty of evidence. However, this finding was not statistically significant.

Our review found no effects of additional vitamin K supplementation, likely explained by the small number of included studies and participants (2 studies, 170 infants). Vitamin K is mostly important in the context of vitamin K deficiency bleeding [107]. Administration of a single intramuscular 1 mg dose of vitamin K for all newborns shortly after birth, regardless of the gestational age or size, is universal clinical practice and the most effective prevention of deficiency-related bleeding [108]. One of the studies in this review investigated alternative doses to the 1 mg dose and found no differences between a 0.3 mg, 0.5 mg, and 1 mg dose [90]. The second study investigated whether additional vitamin K after prolonged antibiotic treatment can prevent antibiotic-related vitamin K deficiency bleeding and found no differences between groups [92].

Vitamin C was shown to reduce the risk of BPD, based on 2 studies including 160 infants, one at high risk of bias, and thus assessed as very low certainty of evidence. Vitamin C has an antioxidant function and aids in regulating inflammation [93].

Besides the 3 studies with few participants investigating vitamin C, we found very little evidence on water-soluble vitamins. One study investigated a combination of vitamin B_12_ and folate supplementation for 4 weeks, finding no effects on NEC, or death in infants born weighing ≤1300 g [96].

Little is known about the status and requirements of water-soluble vitamins in preterm infants. Emerging research points to thiamine as a key nutrient to sustain an adequate growth rate without the risk of lactic acidosis or refeeding syndrome [109–114]. The immaturity of metabolic processes in preterm infants leads to insufficient conversion from nutritive to the bioactive form of vitamins as has been suggested for the conversion from inactive vitamin B_6_ vitamers [115] or decreased activity of vitamin-dependent enzymes such as biotin-dependent carboxylases [116]. The biochemical mechanisms of other vitamins, such as folate and vitamin B_12,_ are intertwined [117,118].

We implemented rigorous methods for this systematic review, leading to a comprehensive and robust overview and evaluation of data, using GRADE to assess the certainty of included evidence. We published a protocol before the inception of the search, detailing the rationale and objective. Any deviations from the protocol were justified and reported. Collecting data on every vitamin supplementation identified important gaps in the evidence regarding the nutritional care for VP and VLBW infants. Few studies were available for vitamins C, B_12_, and folate, and none for other water-soluble vitamins. We also demonstrate a lack of up-to-date evidence on this topic, with half of the included studies published in the 1980s or 1990s, when neonatal care and nutrition were very different.

Included studies used different modes of administration (intramuscular injections, enteral, or intravenous) or a combination. The duration of vitamin supplementation ranged from a single dose to more than 8 weeks, with different regimens such as daily, weekly, or alternating days. Supplementation doses varied widely, and some infants received varying amounts of routine vitamin supplementation as standard nutrition, in addition to the vitamin intervention. This variability presents a substantial challenge in deriving clinical practice recommendations from the findings of our meta-analysis. Many outcomes were inadequately powered, with very few studies and participants included, which may lead to false-positive results. Due to the diversity of participant characteristics and differences in inclusion criteria such as gestational age at birth, weight, age at recruitment, or health status at recruitment, limited individual treatment recommendations can be made from the findings. The certainty of evidence was evaluated as very low for most (76%) outcomes due to inadequate sample size, representativeness of the included studies for the outcome (indirectness), and high risk of bias. Many older studies lack stringency in methodology and reporting to the current scientific standard, reflected in their higher risk of bias assessment.

Even though additional supplementation, especially with vitamins A and E, has been shown to prevent some short-term morbidities, there is little evidence of long-term benefits. Vitamin supplementation, especially intravenous or intramuscular administration of fat-soluble vitamins, is not without risks, and over-supply may have adverse effects. While older studies lack scientific rigour, the methodology of more recent randomised controlled trials is also heterogeneous, making it almost impossible to recommend a specific supplement dose or regimen based on this systematic review.

## Supporting information

S1 File Search strategies. (DOCX)

S2 File Narrative synthesis of studies not included in the meta-analysis. (DOCX)

S1 Table All reports identified in the search. Including bibliographic information and reason for exclusion.
(XLSX)

S2 Table Complete Cochrane Risk of Bias 2 assessment. (XLSX)

S3 Table Table of subgroup analysis. Data are presented as risk ratios [95% confidence interval]. Bold and shaded cells indicate statistical significance. Cells with no data indicate no subgroup analysis was possible. BPD, bronchopulmonary dysplasia; ELBW, extremely low birth weight; IM, intramuscular; IVH, intraventricular haemorrhage; NEC, necrotising enterocolitis; ROP, retinopathy of prematurity.
(XLSX)

S4 Table Consensus data extraction form. Both reviewers (AB and PMB) extracted data in respective extraction forms independently from September to October 2023. Extracted data were compared, discrepancies discussed, and data were entered into a consensus form. Data from the consensus form were entered into RevMan 5.4 for meta-analysis.
(XLSX)

S1 Fig Forest plots for secondary outcomes. (PPTX)

## Abbreviations:

BPD: bronchopulmonary dysplasia
CI: confidence interval
ELBW: extremely low birth weight
GRADE: Grading of Recommendations, Assessment, Development, and Evaluations
IM: intramuscular
IVH: intraventricular haemorrhage
MD: mean difference
NEC: necrotizing enterocolitis
NNT: number needed to treat
RCT: randomized controlled trial
RD: risk difference
ROB: risk of bias
ROP: retinopathy of prematurity
RR: risk ratio
VLBW: very low birth weight
VP: very preterm.
