# Supplementary material for: The effect of vitamin supplementation on neurodevelopmental and clinical outcomes in very low birth weight and very preterm infants: A systematic review and meta-analysis
Source: PLoS One. 2025 Jul 9;20(7):e0327628. doi: 10.1371/journal.pone.0327628 (PMC12240376; doi:10.1371/journal.pone.0327628)
Supplement: S1 File — (DOCX) [file pone.0327628.s001.docx]

**Supplement: Search strategies**

**MEDLINE(via Ovid)**

1 infant, very low birth weight/ or infant, extremely low birth weight/ or infant, premature/ or infant, extremely premature/

2 (((premature or pre-mature or pre-matures or prematures or prematurity or pre-maturity or preterm or preterms or "pre term" or "pre mature") adj3 (baby* or babe or babes or babies or infant* or neonat* or neo-nat* or newborn or "new born" or "newly born")) or "low birthweight" or "low birth weight" or preemie or preemies or premies or premie or VLBW or VLBWI or VLBW-I or VLBWs or LBW or LBWI or LBWs or ELBW or ELBWI or ELBWs or NICU or NICUs).ti,ab,kw.

3 vitamins/ or 24,25-dihydroxyvitamin d 3/ or 25-hydroxyvitamin d 2/ or ascorbic acid/ or calcifediol/ or calcitriol/ or cholecalciferol/ or dihydroxycholecalciferols/ or tocopherols/ or tocotrienols/ or vitamin a/ or vitamin d/ or vitamin e/ or vitamin k/ or vitamin k 1/ or vitamin k 3/ or alpha-tocopherol/ or beta-tocopherol/ or gamma-tocopherol/ or beta carotene/ or biotin/ or folic acid/ or formyltetrahydrofolates/ or hydroxocobalamin/ or niacin/ or niacinamide/ or pantothenic acid/ or pyridoxal/ or pyridoxal phosphate/ or pyridoxamine/ or pyridoxine/ or riboflavin/ or tetrahydrofolates/ or thiamine/ or thiamine monophosphate/ or thiamine pyrophosphate/ or thiamine triphosphate/ or vitamin b 12/ or vitamin b 6/

4 ("vitamin A" or retinol or retinoid* or retinal).ti,ab,kw.

5 ("vitamin D" or calcitriol or 1,25-dihydroxyvitamin or 25-dihydroxyvitamin).ti,ab,kw.

6 ("vitamin E" or tocopherol* or tocotrienol*).ti,ab,kw.

7 ("vitamin K" or phylloquinone or menaquinone).ti,ab,kw.

8 ("Vitamin B1" or "vitamin b 1" or thiamine or thiamin).ti,ab,kw.

9 ("Vitamin B2" or "vitamin b 2" or riboflavin).ti,ab,kw.

10 ("Vitamin B3" or "vitamin b 3" or niacin).ti,ab,kw.

11 ("Vitamin B5" or "vitamin b 5" or "pantothenic acid").ti,ab,kw.

12 ("Vitamin B6" or "vitamin b 6" or pyridoxine or pyridoxal or pyridoxamine).ti,ab,kw.

13 ("Vitamin B7" or "vitamin b 7" or biotin).ti,ab,kw.

14 ("Vitamin B9" or "vitamin b 9" or folate or folic acid).ti,ab,kw.

15 ("Vitamin B12" or "vitamin b 12" or cobalamin).ti,ab,kw.

16 ("Vitamin C" or "ascorbic acid" or ascorbate).ti,ab,kw.

17 1 or 2

18 3 or 4 or 5 or 6 or 7 or 8 or 9 or 10 or 11 or 12 or 13 or 14 or 15 or 16

19 17 and 18

20 randomi*ed controlled trial.pt.

21 controlled clinical trial.pt.

22 randomi*ed.ti,ab.

23 placebo.ti,ab.

24 drug therapy.fs.

25 randomly.ti,ab.

26 groups.ti,ab.

27 trial.ti,ab.

28 20 or 21 or 22 or 23 or 24 or 25 or 26 or 27

29 (quasirandom* or quasi-random* or random*).ti,ab,kw,kf.

30 (control* adj2 (group? or trial? or study)).ti,ab,kw,kf.

31 29 or 30

32 exp animals/ not humans/

33 (or/28,31) not 32

34 19 and 33

**EMBASE (via Ovid)**

1 prematurity/

2 very low birth weight/

3 (((premature or pre-mature or pre-matures or prematures or prematurity or pre-maturity or preterm or preterms or "pre term" or "pre mature") adj3 (baby* or babe or babes or babies or infant* or neonat* or neo-nat* or newborn or "new born" or "newly born")) or "low birthweight" or "low birth weight" or preemie or preemies or premies or premie or VLBW or VLBWI or VLBW-I or VLBWs or LBW or LBWI or LBWs or ELBW or ELBWI or ELBWs or NICU or NICUs).ti,ab,kw.

4 vitamin/ or alpha tocopherol plus ergocalciferol plus ergocalciferol plus retinol palmitate/ or ascorbic acid/ or carotenoid/ or multivitamin/ or provitamin/ or tocopherol/ or vitamin b group/ or vitamin d/ or vitamin k group/ or vitamin mixture/

5 ("vitamin A" or retinol or retinoid* or retinal).ti,ab,kw.

6 ("vitamin D" or calcitriol or 1,25-dihydroxyvitamin or 25-dihydroxyvitamin).ti,ab,kw.

7 ("vitamin E" or tocopherol* or tocotrienol*).ti,ab,kw.

8 ("vitamin K" or phylloquinone or menaquinone).ti,ab,kw.

9 ("vitamin B1" or "vitamin B 1" or thiamine or thiamin).ti,ab,kw.

10 ("vitamin B2" or "vitamin B 2" or riboflavin).ti,ab,kw.

11 ("vitamin B3" or "vitamin B 3" or niacin).ti,ab,kw.

12 ("vitamin B5" or "vitamin B 5" or "pantothenic acid").ti,ab,kw.

13 ("vitamin B6" or "vitamin B 6" or pyridoxine or pyridoxal or pyridoxamine).ti,ab,kw.

14 ("vitamin B7" or "vitamin B 7" or biotin).ti,ab,kw.

15 ("vitamin B9" or "vitamin B 9" or folate or folic acid).ti,ab,kw.

16 ("vitamin B12" or "vitamin B 12" or cobalamin).ti,ab,kw.

17 ("vitamin C" or "ascorbic acid").ti,ab,kw.

18 1 or 2 or 3

19 4 or 5 or 6 or 7 or 8 or 9 or 10 or 11 or 12 or 13 or 14 or 15 or 16 or 17

20 18 and 19

21 randomized controlled trial/

22 controlled clinical trial/

23 random$.ti,ab.

24 randomization/

25 placebo.ti,ab.

26 (open adj label).ti,ab.

27 ((double or single or doubly or singly) adj (blind or blinded or blindly)).ti,ab.

28 double blind procedure/

29 parallel group$1.ti,ab.

30 (crossover or cross over).ti,ab.

31 ((assign$ or match or matched or allocation) adj5 (alternate or group$1 or intervention$1 or patient$1 or subject$1 or participant$1)).ti,ab.

32 (controlled adj7 (study or design or trial)).ti,ab.

33 (quasirandom* or quasi-random* or random*).ti,ab,kw,kf.

34 (control* adj2 group?).ti,ab,kw,kf.

35 21 or 22 or 23 or 24 or 25 or 26 or 27 or 28 or 29 or 30 or 31 or 32 or 33 or 34

36 (exp animals/ or exp invertebrate/ or animal experiment/ or animal model/ or animal tissue/ or animal cell/ or nonhuman/) and (human/ or normal human/ or human cell/)

37 35 not 36

38 20 and 37

**CINAHL (via EBSCO)**

S1 (MH “Infant, Very Low Birth Weight”) OR (MH “Infant, Premature”)

S2 TI ((((premature or pre-mature or prematures or prematurity or pre-maturity or preterm or preterms or “pre term” or “pre mature”) adj3 (baby* or babe or babes or babies or infant* or neonat* or neo-nat* or newborn or “new-born” or “newly born”)) or “low birthweight” or “low birth weight” or preemie or preemies or premies or premie or VLBW or VLBWI or VLBW-I or VLBWs or LBW or LBWI or LBWs or ELBWI or WLBW or ELBWs or NICU or NICUs)) OR AB ((((premature or pre-mature or prematures or prematurity or pre-maturity or preterm or preterms or “pre term” or “pre mature”) adj3 (baby* or babe or babes or babies or infant* or neonat* or neo-nat* or newborn or “new-born” or “newly born”)) or “low birthweight” or “low birth weight” or preemie or preemies or premies or premie or VLBW or VLBWI or VLBW-I or VLBWs or LBW or LBWI or LBWs or ELBWI or ELBW or ELBWs or NICU or NICUs))

S3 (MH "Pantothenic Acid") OR (MH "Vitamin K") OR (MH "Vitamin E") OR (MH "Vitamin D") OR (MH "Calcitriol") OR (MH "Cholecalciferol") OR (MH "Ergocalciferols") OR (MH "Biotin") OR (MH "Folic Acid") OR (MH "Niacin") OR (MH "Pyridoxine") OR (MH "Thiamine") OR (MH "Vitamin B12") OR (MH "Vitamin A") OR (MH "Beta Carotene") OR (MH "Ascorbic Acid") OR (MH "Vitamin B Complex")

S4 TI (("vitamin A" or retinol or retinoid* or retinal)) OR AB (("vitamin A" or retinol or retinoid* or retinal))

S5 TI (("vitamin D" or calcitriol or 1,25-dihydroxyvitamin or 25-dihydroxyvitamin)) OR AB (("vitamin D" or calcitriol or 1,25-dihydroxyvitamin or 25-dihydroxyvitamin))

S6 TI (("vitamin E" or tocopherol" or tocotrienol*)) OR AB (("vitamin E" or tocopherol or tocotrienol*))

S7 TI (("vitamin K" or phylloquinone or menaquinone)) OR AB ("vitamin K" or

phylloquinone or menaquinone))

S8 TI (("Vitamin B1" or "vitamin b 1" or thiamine or thiamin)) OR AB (("Vitamin B1" or "vitamin b 1" or thiamine or thiamin))

S9 TI (("Vitamin B2" or "vitamin b 2" or riboflavin)) OR AB (("Vitamin B2" or "vitamin b 2" or riboflavin))

S10 TI (("Vitamin B3" or "vitamin b 3" or niacin)) OR AB (("Vitamin B3" or "vitamin b 3" or niacin))

S11 TI (("Vitamin B5" or "vitamin b 5" or "pantothenic acid")) OR AB (("Vitamin B5" or "vitamin b 5" or "pantothenic acid"))

S12 TI (("Vitamin B6" or "vitamin b 6" or pyridoxine or pyridoxal or pyridoxamine)) OR AB (("Vitamin B6" or "vitamin b 6" or pyridoxine or pyridoxal or pyridoxamine))

S13 TI (("Vitamin B7" or "vitamin b 7" or biotin)) OR AB (("Vitamin B7" or "vitamin b 7" or biotin))

S14 TI (("Vitamin B9" or "vitamin b 9" or folate or folic acid)) OR AB (("Vitamin B9" or "vitamin b 9" or folate or folic acid))

S15 TI (("Vitamin B12" or "vitamin b 12" or cobalamin)) OR AB (("Vitamin B12" or "vitamin b 12" or cobalamin))

S16 TI (("Vitamin C" or "ascorbic acid")) OR AB (("Vitamin C" or "ascorbic acid"))

S17 S1 or S2

S18 S3 or S4 or S5 or S6 or S7 or S8 or S9 or S10 or S11 or S12 or S13 or S14 or S15 or S16

S19 (MH randomized controlled trials) or (MH double-blind studies) or (MH single-blind studies) or (MH random assignment) or (MH pretest-posttest design) or (MH cluster sample)

S20 TI (randomised OR randomized)

S21 AB (random*)

S22 TI (trial)

S23 MH (sample size) AND AB (assigned OR allocated OR control)

S24 MH (placebos)

S25 PT (randomized controlled trial)

S26 AB (control W5 group)

S27 MH (crossover design) OR MH (comparative studies)

S28 AB (cluster W3 RCT)

S29 MH animals+

S30 MH (animal studies)

S31 TI (animal model")

S32 S29 or S30 or S31

S33 MH (human)

S34 S32 not S33

S35 S19 or S20 or S21 or S22 or S23 or S24 or S25 or S26 or S27 or S28

S36 S35 not S34

S37 S17 and S18 and S36

**CENTRAL (via Cochrane)**

#1 MeSH descriptor: [Infant, Premature] explode all trees

#2 MeSH descriptor: [Infant, Very Low Birth Weight] explode all trees

#3 ((premature or pre-mature or pre-matures or prematures or prematurity or pre-maturity or preterm or preterms or "pre term" or "pre mature") adj3 (baby* or babe or babes or babies or infant* or neonat* or neo-nat* or newborn or "new born" or "newly born")) or "low birthweight" or "low birth weight" or preemie or preemies or premies or premie or VLBW or VLBWI or VLBW-I or VLBWs or LBW or LBWI or LBWs or ELBW or ELBWI or ELBWs or NICU or NICUs

#4 MeSH descriptor: [Vitamins] explode all trees

#5 MeSH descriptor: [Ascorbic Acid] explode all trees

#6 MeSH descriptor: [Carotenoids] 1 tree(s) exploded

#7 MeSH descriptor: [Vitamin D] explode all trees

#8 MeSH descriptor: [Vitamin E] explode all trees

#9 MeSH descriptor: [Vitamin K] explode all trees

#10 MeSH descriptor: [Thiamine] explode all trees

#11 MeSH descriptor: [Riboflavin] explode all trees

#12 MeSH descriptor: [Niacin] explode all trees

#13 MeSH descriptor: [Pantothenic Acid] 1 tree(s) exploded

#14 MeSH descriptor: [Vitamin B 6] explode all trees

#15 MeSH descriptor: [Folic Acid] explode all trees

#16 MeSH descriptor: [Biotin] explode all trees

#17 MeSH descriptor: [Vitamin B 12] explode all trees

#18 #1 or #2 or #3

#19 (("vitamin A" or retinol or retinoid* or retinal)):ti,ab,kw

#20 (("vitamin d" or calcitriol or "1,25-dihydroxyvitamin" or "25-dihydroxyvitamin")):ti,ab,kw

#21 (("vitamin E" or tocopherol* or tocotrienol*)):ti,ab,kw

#22 (("vitamin K" or phylloquinone or menaquinone)):ti,ab,kw

#23 (("Vitamin B1" or "vitamin b 1" or thiamine or thiamin)):ti,ab,kw

#24 (("Vitamin B2" or "vitamin b 2" or riboflavin)):ti,ab,kw

#25 (("Vitamin B3" or "vitamin b 3" or niacin)):ti,ab,kw

#26 (("Vitamin B5" or "vitamin b 5" or "pantothenic acid")):ti,ab,kw

#27 (("Vitamin B6" or "vitamin b 6" or pyridoxine or pyridoxal or pyridoxamine)):ti,ab,kw

#28 (("Vitamin B7" or "vitamin b 7" or biotin)):ti,ab,kw

#29 (("Vitamin B9" or "vitamin b 9" or folate or folic acid)):ti,ab,kw

#30 (("Vitamin B12" or "vitamin b 12" or cobalamin)):ti,ab,kw

#31 (("Vitamin C" or "ascorbic acid")):ti,ab,kw

#32 #4 or #5 or #6 or #7 or #8 or #9 or #10 or #11 or #12 or #13 or #14 or #15 or #16 or #17 or #19 or #20 or #21 or #22 or #23 or #24 or #25 or #26 or #27 or #28 or #29 or #30 or #31

#33 #18 and #32

**ClinicalTrials.gov**

vitamin | preterm or premature

**The WHO International Clinical Trials Registry Platform (ICTRP)**

(multivitamin or "vitamin A" or retinol or retinoid* or retinal or "vitamin D" or calcitriol or 1,25-dihydroxyvitamin or 25-dihydroxyvitamin or "vitamin E" or tocopherol* or tocotrienol* or "vitamin K" or phylloquinone or menaquinone or "Vitamin B1" or "vitamin b 1" or thiamine or thiamin or "Vitamin B2" or "vitamin b 2" or riboflavin or "Vitamin B3" or "vitamin b 3" or niacin or "Vitamin B5" or "vitamin b 5" or "pantothenic acid" or "Vitamin B6" or "vitamin b 6" or pyridoxine or pyridoxal or pyridoxamine or "Vitamin B7" or "vitamin b 7" or biotin or "Vitamin B9" or "vitamin b 9" or folate or “folic acid” or "Vitamin B12" or "vitamin b 12" or cobalamin or "Vitamin C" or "ascorbic acid") and (premature or pre-mature or pre-matures or prematures or prematurity or pre-maturity or preterm or preterms or "pre term" or "pre mature" or "low birthweight" or "low birth weight" or preemie or preemies or premies or premie or VLBW or VLBWI or VLBW-I or VLBWs or LBW or LBWI or LBWs or ELBW or ELBWI or ELBWs or NICU or NICUs)

**The Australian and New Zealand Clinical Trials Registry**

(preterm or premature or "low birthweight") and (vitamin or vitamins or multivitamin)
