# Supplementary material for: The effect of vitamin supplementation on neurodevelopmental and clinical outcomes in very low birth weight and very preterm infants: A systematic review and meta-analysis
Source: PLoS One. 2025 Jul 9;20(7):e0327628. doi: 10.1371/journal.pone.0327628 (PMC12240376; doi:10.1371/journal.pone.0327628)
Supplement: S2 File — (DOCX) [file pone.0327628.s002.docx]

**Studies not included in the meta-analysis**

Three studies provided only qualitative data; hence, they were not eligible for meta-analysis, and 4 studies provided quantitative data only for some outcomes.

The cohorts included in Koo 1995a [1] and Koo 1995b [2] overlapped, and the studies were thus excluded from the meta-analysis. Both studies found no effect of vitamin A or D on growth measured as weight, length, and head circumference gain from birth to discharge or weight of 2000 g.

Calisici 2014 [3] found no statistically significant effect of enteral supplementation of 30000 IU.kg^-1^.week^-1^ vitamin A for 6 weeks on late-onset sepsis, severe IVH, ROP, BPD, or mortality. Pearson 1992 [4] reported no differences in incidences of IVH, NEC, or sepsis between study groups. One group received 2000 IU of vitamin A intramuscularly every second day for 4 weeks, while the comparator group received placebo or sham injections.

Sinha 1987 [5] investigated the effect of 3 intramuscular doses of 20 mg.kg^-1^ vitamin E during the first 3 days after birth. The study reported that neither early- and late-onset culture-proven sepsis nor mortality differed between the groups. Another study investigated the effect of a single dose of 50 IU.kg^-1^ vitamin E within 4 hours after birth and reported no effect of supplementation on NEC, late-onset sepsis, severe IVH, or death during the first 2 weeks after birth. These outcomes were recorded as adverse events, and the study was not adequately powered for these outcomes [6]. Watts 1991 [7] compared the effects of 25 IU enteral vitamin E daily for 6 weeks versus placebo and found no association between supplementation and incidence of NEC or sepsis.

Growth was reported differently in every study, as daily weight gain over a period, weight at discharge relative to birth weight, or z-scores. Therefore, these data could not be summarised in a meta-analysis. No study found differences between groups in any growth parameters for any vitamin supplementation. For 2 studies investigating vitamin E supplementation, growth velocity (g.kg^-1^.d^-1^), according to Patel [8], could be calculated. For Pathak 2003, the growth velocity from birth to discharge (mean 69 days) was calculated as 11.7 g.kg^-1^.d^-1^ in the exposure group and 11.8 g.kg^-1^.d^-1^ in the control group [9]. For Tripathi 2011, growth velocity from birth to day 21 was calculated as 1.9 g.kg^-1^.d^-1^ in the exposure group and 3.2 g.kg^-1^.d^-1^ in the control group [10]. A meta-analysis was not performed because individual participant data was not available to calculate standard deviations.

Anaemia was also reported differently, as number of transfusions per infants, or number of infants who required transfusions. One study supplementing vitamin E [9], two studies supplementing vitamin C [11, 12] (but using different outcome measures), and one study supplementing vitamin B_12_ and folate [13] reported on anaemia outcomes, but none showed any effect.

1. Koo WW, Krug-Wispe S, Succop P, Tsang RC, Neylan M. Effect of different vitamin A intakes on very-low-birth-weight infants. Am J Clin Nutr. 1995;62(6):1216-20. doi: 10.1093/ajcn/62.6.1216.

2. Koo WW, Krug-Wispe S, Neylan M, Succop P, Oestreich AE, Tsang RC. Effect of three levels of vitamin D intake in preterm infants receiving high mineral-containing milk. Journal of Pediatric Gastroenterology & Nutrition. 1995;21(2):182-9. doi: 10.1097/00005176-199508000-00010.

3. Calisici E, Yarci E, Degirmencioglu H, Oncel M, Oguz S, Uras N, et al. The effects of early oral vitamin a treatment on the prevention of bronchopulmonary displasia in the low birth weight infants. Archives of Disease in Childhood. 2014;99(Suppl 2):A494. doi: 10.1136/archdischild-2014-307384.1371.

4. Pearson E, Bose C, Snidow T, Ransom L, Young T, Bose G, et al. Trial of vitamin A supplementation in very low birth weight infants at risk for bronchopulmonary dysplasia. Journal of Pediatrics. 1992;121(3):420-7. doi: 10.1016/S0022-3476(05)81800-1.

5. Sinha S, Toner N, Davies J, Bogle S, Chiswick M. Vitamin E supplementation reduces frequency of periventricular haemorrhage in very preterm babies. Lancet. 1987;329(8531):466-71. doi: 10.1016/S0140-6736(87)92087-3.

6. Bell EF, Hansen NI, Brion LP, Ehrenkranz RA, Kennedy KA, Walsh MC, et al. Serum tocopherol levels in very preterm infants after a single dose of vitamin E at birth. Pediatrics. 2013;132(6):e1626-e33. doi: 10.1542/peds.2013-1684.

7. Watts JL, Milner R, Zipursky A, Paes B, Ling E, Gill G, et al. Failure of supplementation with vitamin E to prevent bronchopulmonary dysplasia in infants less than 1,500 g birth weight. European Respiratory Journal. 1991;4(2):188-90.

8. Patel AL, Engstrom JL, Meier PP, Kimura RE. Accuracy of methods for calculating postnatal growth velocity for extremely low birth weight infants. Pediatrics. 2005;116(6):1466-73. doi: 10.1542/peds.2004-1699.

9. Pathak A. Effects of vitamin E supplementation during erythropoietin treatment of the anaemia of prematurity. Archives of disease in childhood Fetal and neonatal edition. 2003;88(4):324F-8. doi: 10.1136/fn.88.4.f324.

10. Tripathi S, Mishra TK, Mathur NB. Vitamin E supplementation in exclusively breastfed VLBW infants. Indian Pediatrics. 2011;48(11):889-91. doi: 10.1007/s13312-011-0136-4.

11. Bass WT, Malati N, Castle MC, White LE. Evidence for the safety of ascorbic acid administration to the premature infant. American journal of perinatology. 1998;15(2):133-40. doi: 10.1055/s-2007-993913.

12. Doyle J, Vreman HJ, Stevenson DK, Brown EJ, Schmidt B, Paes B, et al. Does vitamin C cause hemolysis in premature newborn infants? Results of a multicenter double-blind, randomized, controlled trial. Journal of Pediatrics. 1997;130(1):103-9. doi: 10.1016/s0022-3476(97)70317-2.

13. Haiden N, Klebermass K, Cardona F, Schwindt J, Berger A, Kohlhauser-Vollmuth C, et al. A randomized, controlled trial of the effects of adding vitamin B12 and folate to erythropoietin for the treatment of anemia of prematurity. Pediatrics. 2006;118(1):180-8. doi: 10.1542/peds.2005-2475
