# Supplementary material for: The effect of vitamin supplementation on neurodevelopmental and clinical outcomes in very low birth weight and very preterm infants: A systematic review and meta-analysis
Source: PLoS One. 2025 Jul 9;20(7):e0327628. doi: 10.1371/journal.pone.0327628 (PMC12240376; doi:10.1371/journal.pone.0327628)
Supplement: S1 Fig — (PPTX) [file pone.0327628.s007.pptx]

## Slide 1
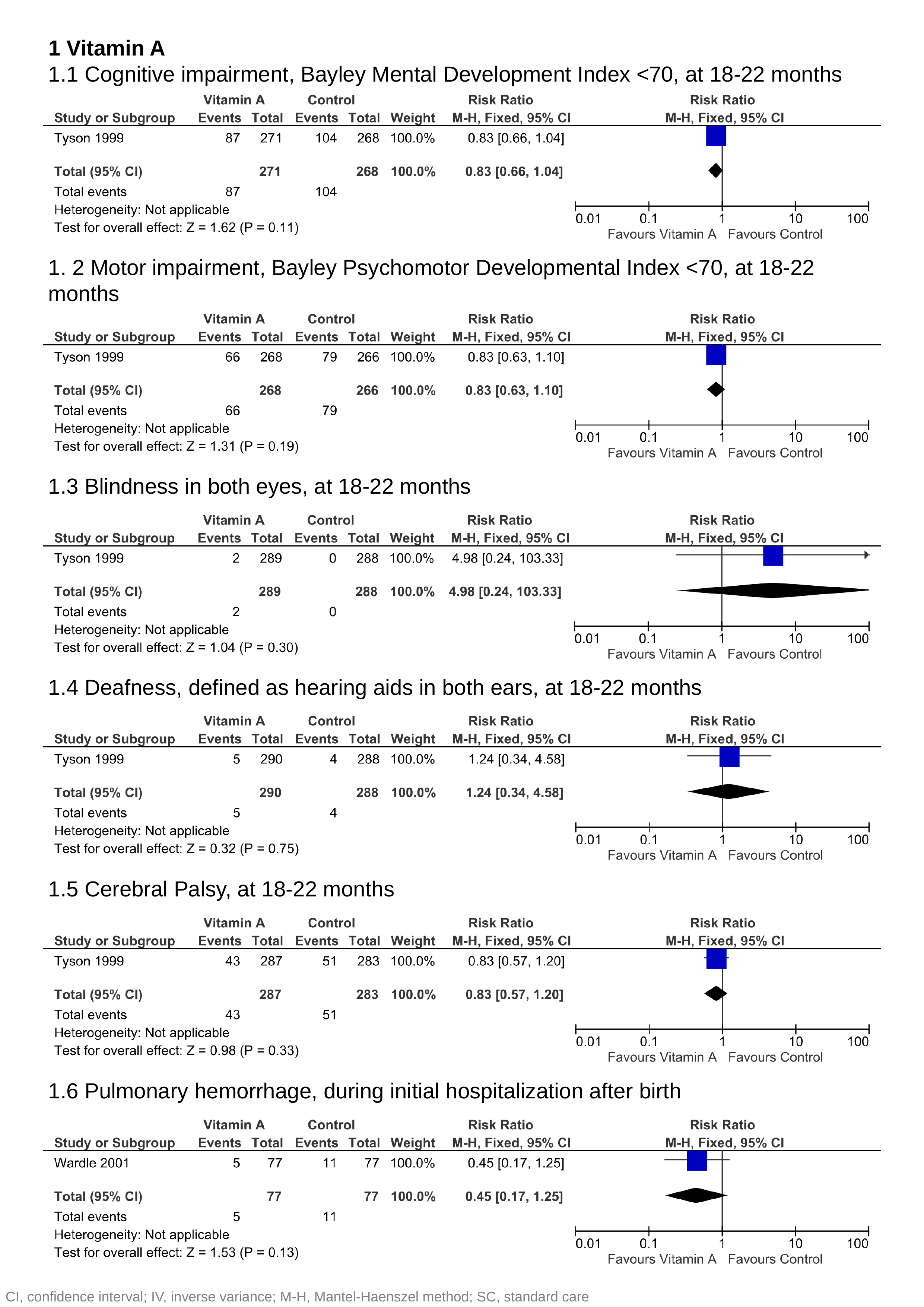

1 Vitamin A
1.1 Cognitive impairment, Bayley Mental Development Index <70, at 18-22 months
1. 2 Motor impairment, Bayley Psychomotor Developmental Index <70, at 18-22 months
1.3 Blindness in both eyes, at 18-22 months
1.4 Deafness, defined as hearing aids in both ears, at 18-22 months
1.5 Cerebral Palsy, at 18-22 months
1.6 Pulmonary hemorrhage, during initial hospitalization after birth
CI, confidence interval; IV, inverse variance; M-H, Mantel-Haenszel method; SC, standard care

## Slide 2
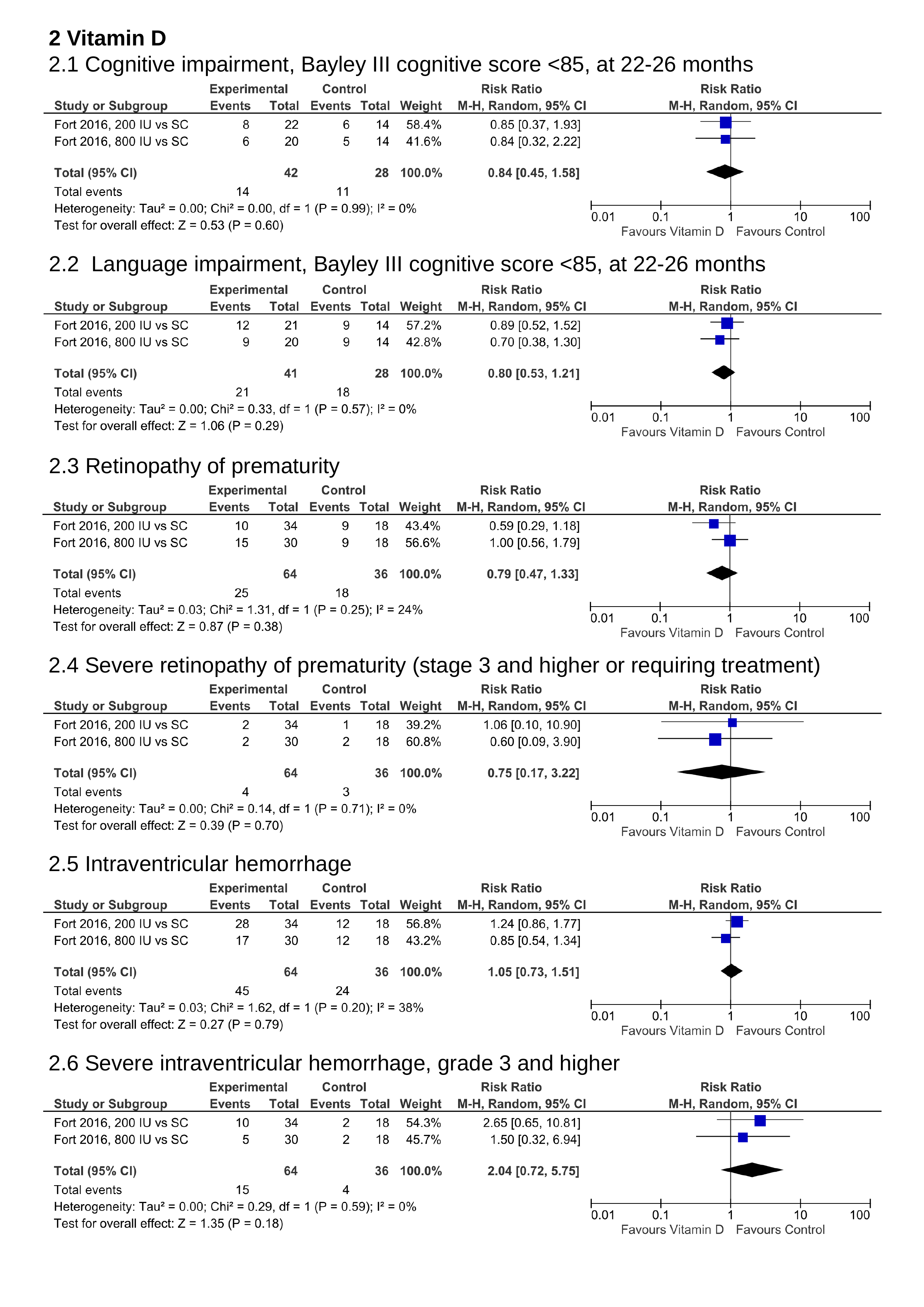

2 Vitamin D
2.1 Cognitive impairment, Bayley III cognitive score <85, at 22-26 months
2.2 Language impairment, Bayley III cognitive score <85, at 22-26 months
2.3 Retinopathy of prematurity
2.4 Severe retinopathy of prematurity (stage 3 and higher or requiring treatment)
2.5 Intraventricular hemorrhage
2.6 Severe intraventricular hemorrhage, grade 3 and higher

## Slide 3
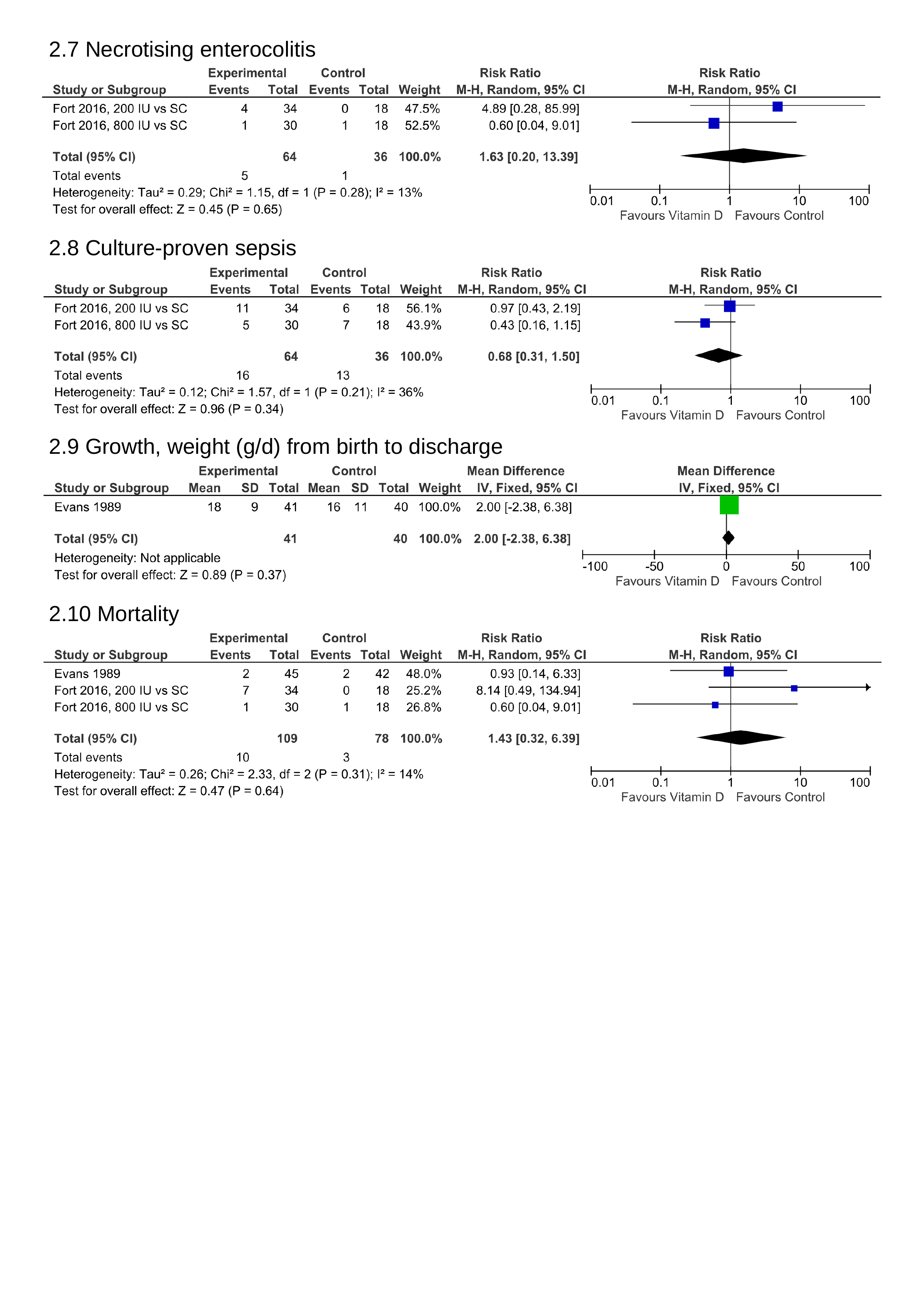

2.7 Necrotising enterocolitis
2.8 Culture-proven sepsis
2.9 Growth, weight (g/d) from birth to discharge
2.10 Mortality

## Slide 4
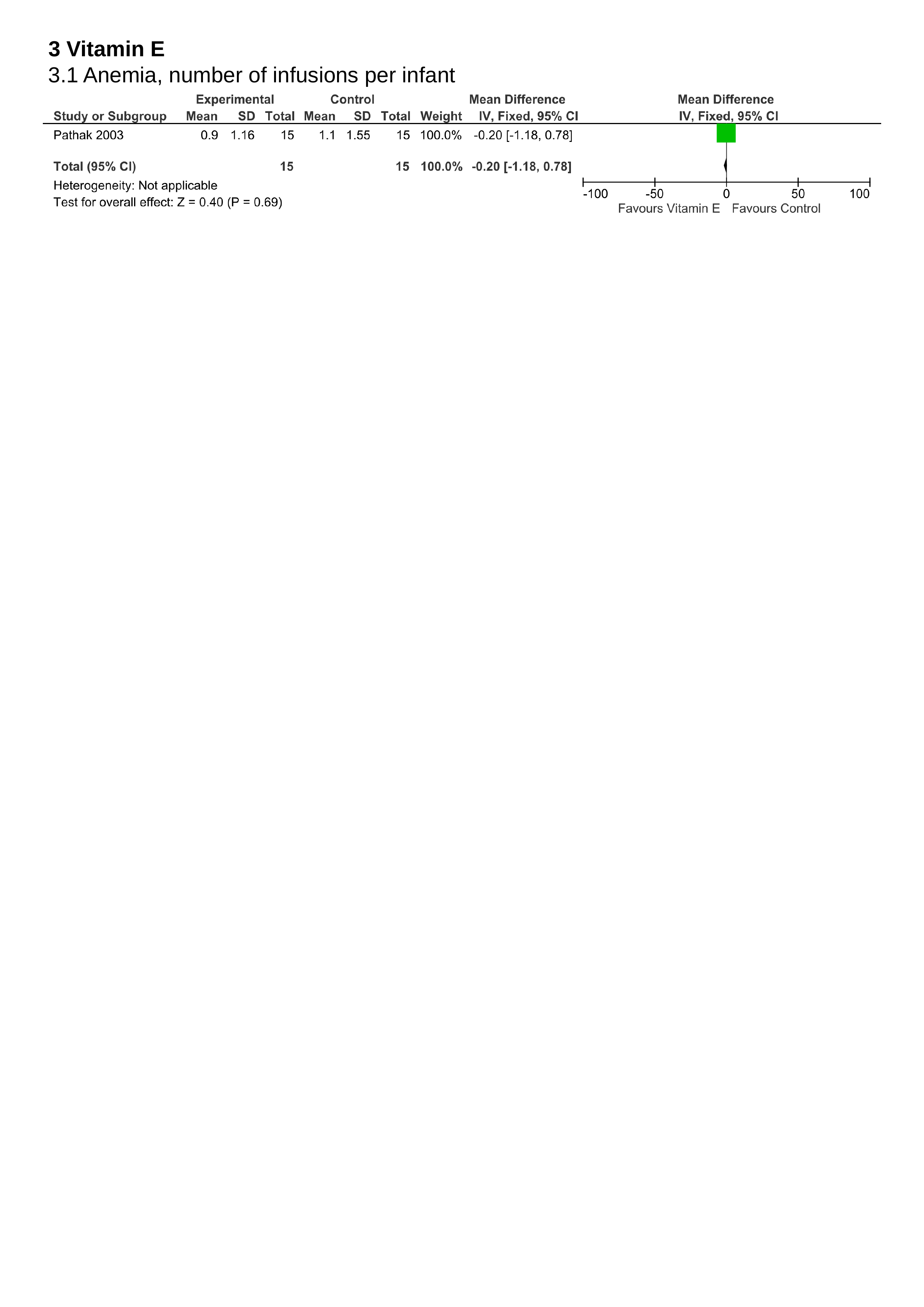

3 Vitamin E
3.1 Anemia, number of infusions per infant

## Slide 5
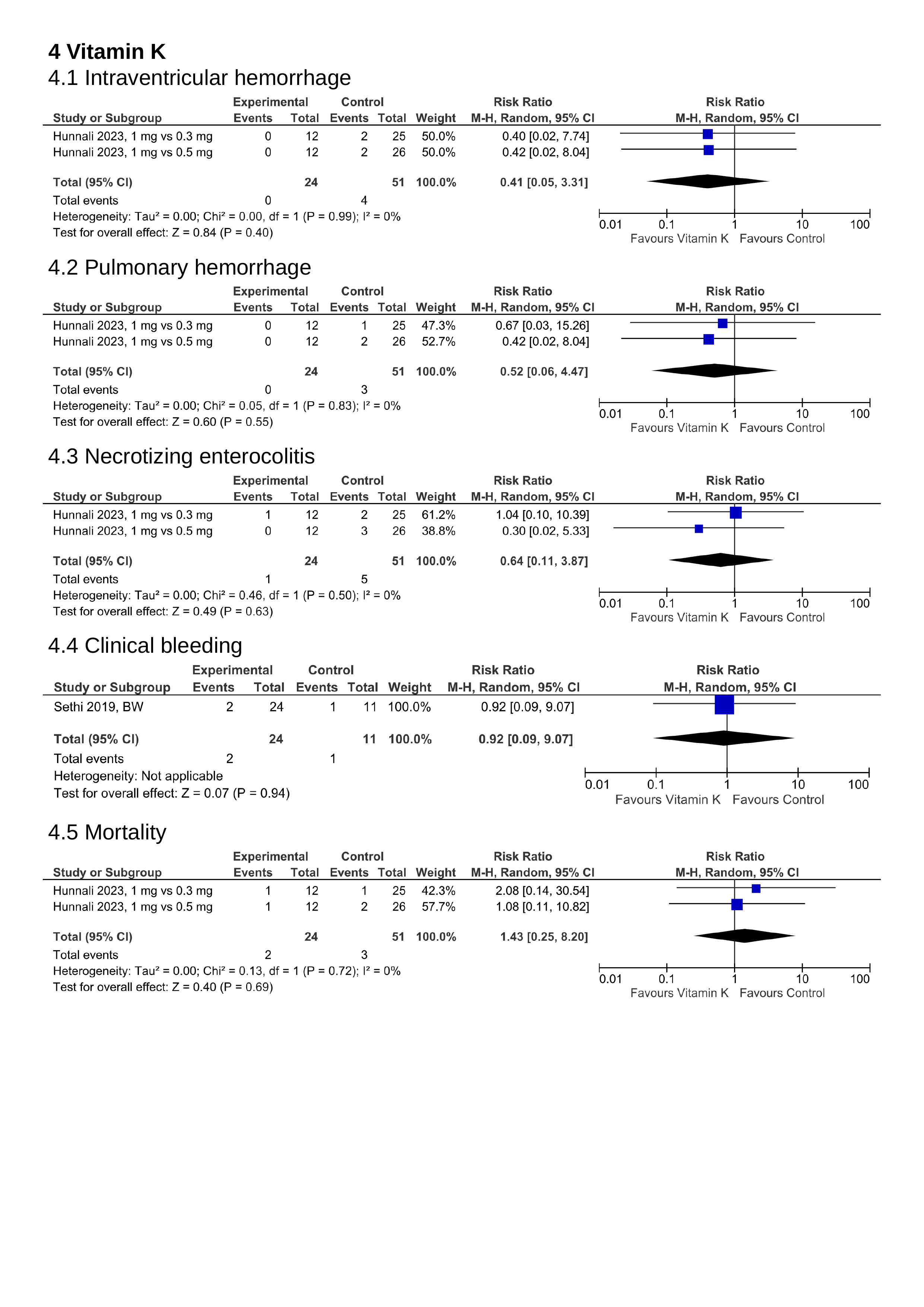

4 Vitamin K
4.1 Intraventricular hemorrhage
4.2 Pulmonary hemorrhage
4.3 Necrotizing enterocolitis
4.4 Clinical bleeding
4.5 Mortality

## Slide 6
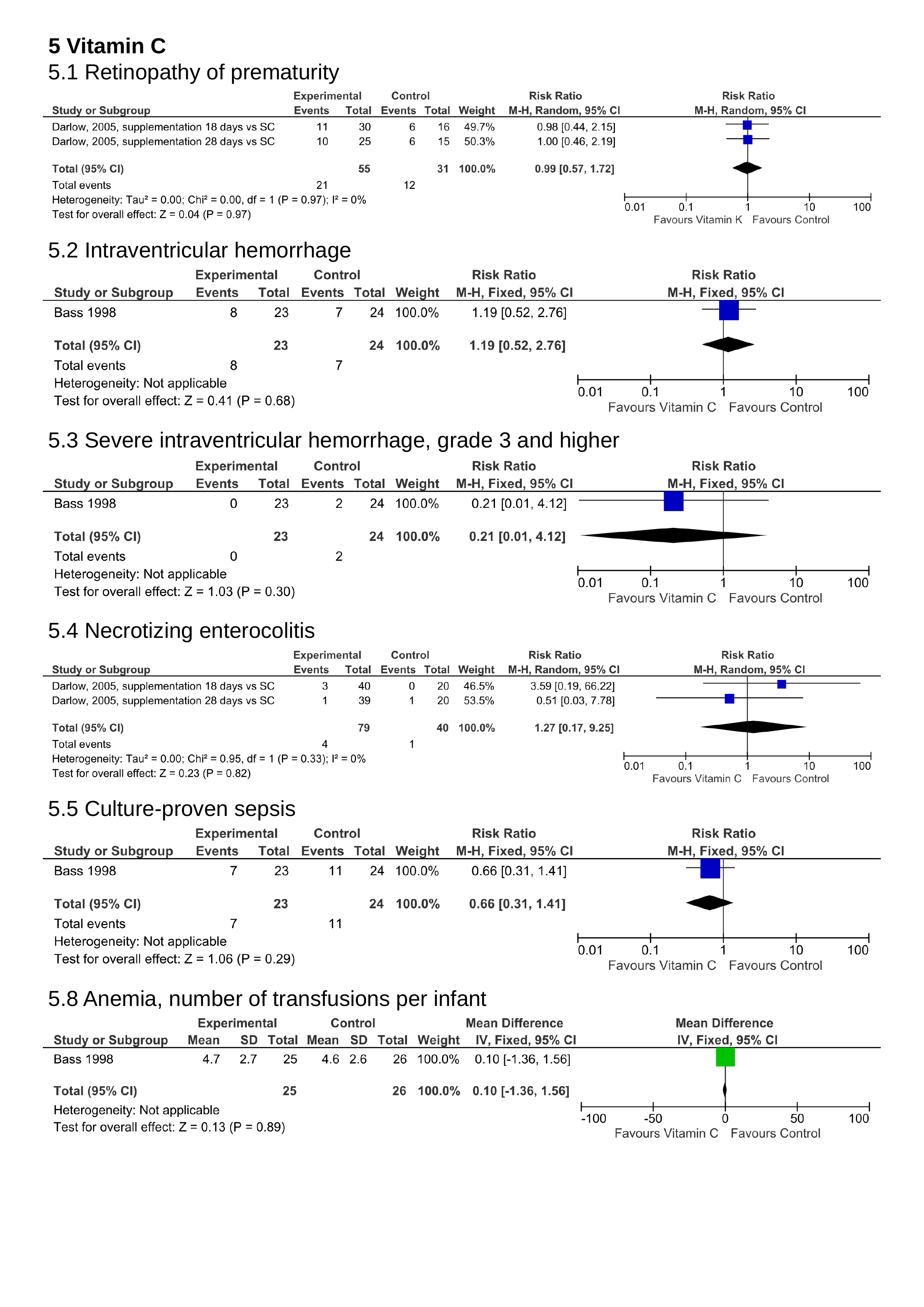

5 Vitamin C
5.1 Retinopathy of prematurity
5.2 Intraventricular hemorrhage
5.3 Severe intraventricular hemorrhage, grade 3 and higher
5.4 Necrotizing enterocolitis
5.5 Culture-proven sepsis
5.8 Anemia, number of transfusions per infant

## Slide 7
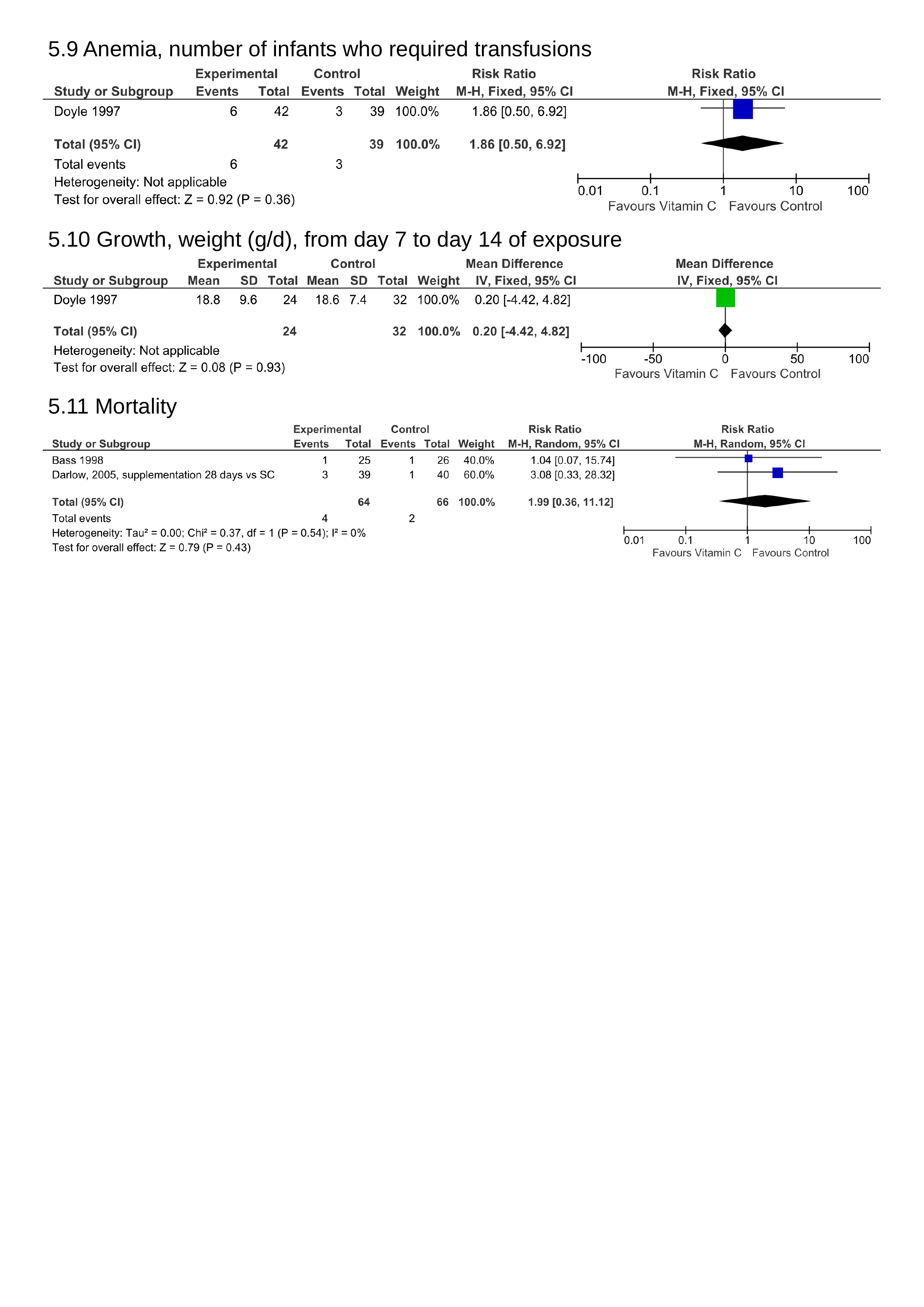

5.9 Anemia, number of infants who required transfusions
5.10 Growth, weight (g/d), from day 7 to day 14 of exposure
5.11 Mortality

## Slide 8
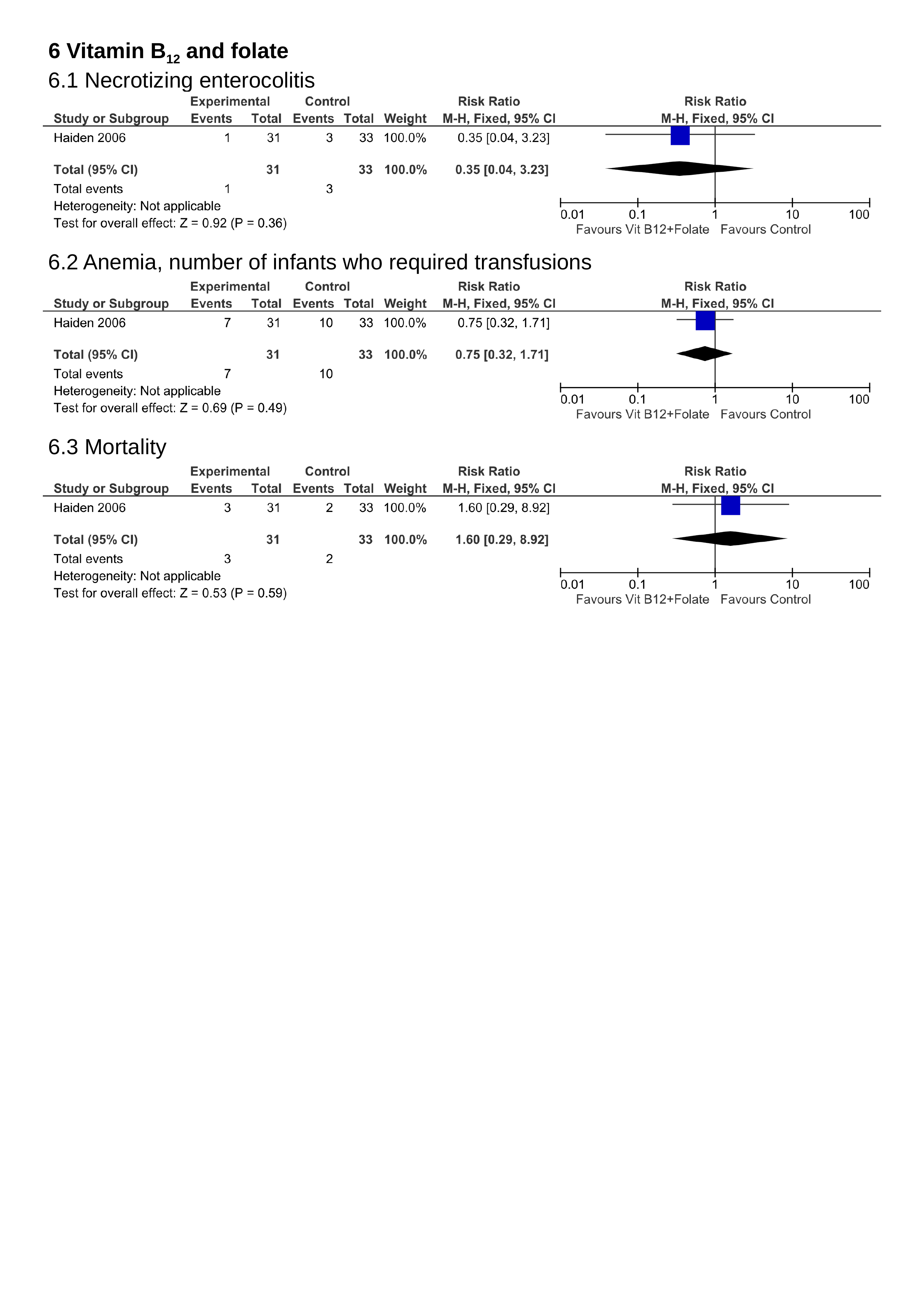

6 Vitamin B12 and folate
6.1 Necrotizing enterocolitis
6.2 Anemia, number of infants who required transfusions
6.3 Mortality
